# Evidence for the Rapid and Divergent Evolution of Mycoplasmas: Structural and Phylogenetic Analysis of Enolases

**DOI:** 10.3389/fmolb.2021.811106

**Published:** 2022-01-25

**Authors:** Rong Chen, Lin Zhao, Rong Gan, Zhixin Feng, Chenxi Cui, Xing Xie, Fei Hao, Zhenzhen Zhang, Li Wang, Tingting Ran, Weiwu Wang, Shuijun Zhang, Yufeng Li, Wei Zhang, Maoda Pang, Qiyan Xiong, Guoqing Shao

**Affiliations:** ^1^ College of Veterinary Medicine, Nanjing Agricultural University, Nanjing, China; ^2^ Institute of Veterinary Medicine, Jiangsu Academy of Agricultural Sciences, Nanjing, China; ^3^ National Laboratory of Biomacromolecules, CAS Center for Excellence in Biomacromolecules, Institute of Biophysics, Chinese Academy of Sciences, Beijing, China; ^4^ Key Laboratory of Agricultural and Environmental Microbiology, College of Life Sciences, Ministry of Agriculture, Nanjing Agricultural University, Nanjing, China; ^5^ College of Life Sciences, Nanjing Agricultural University, Nanjing, China; ^6^ Key Lab of Animal Bacteriology of Ministry of Agriculture, OIE Reference Lab for Swine Streptococcosis, College of Veterinary Medicine, Nanjing Agricultural University, Nanjing, China; ^7^ State Key Laboratory Cultivation Base of MOST, Institute of Food Safety and Nutrition, Jiangsu Academy of Agricultural Sciences, Nanjing, China

**Keywords:** crstal structure, enolase, divergent evolution, mycoplasma, mollicutes

## Abstract

Mycoplasmas are a group of prokaryotes without cell walls that have evolved through several rounds of degenerative evolution. With a low cell DNA G + C content and definitively long genetic lineages, mycoplasmas are thought to be in a state of rapid evolution. However, little associated evidence has been provided. Enolase is a key enzyme in glycolysis that is widely found in all species from the three domains, and it is evolutionarily conserved. In our previous studies, enolase acted as a virulence factor and participated in cell-surface adhesion in *Mycoplasma hyopneumoniae*. Furthermore, unique loop regions were first found in the crystal structure of Mhp Eno. Here, enolase structures from *Mycoplasma pneumoniae* and *Mycoplasma bovis* were determined. An extra helix 7 is specific and conservatively found in almost all *mycoplasma* enolases, as confirmed by crystal structures and sequence alignment. Particular motifs for helix 7, which is composed of F-K/G-K-L/F-K-X-A-I, have been proposed and could be regarded as molecular markers. To our surprise, the genetic distances between any two *mycoplasma* enolases were obviously longer than those between the two corresponding species themselves, indicating divergent evolution of *mycoplasma* enolases, whereas no horizontal gene transfer was detected in *mycoplasma* enolase genens. Furthermore, different evolutionary patterns were adopted by different loop regions of *mycoplasma* enolase. Enolases from different *Mycoplasma* species also showed different affinities for PLG and fibronectin. Our results indicate the rapid and divergent evolution of *mycoplasma* enolase and mycoplasmas. This study will also aid understanding the independent evolution of *Mycoplasma* species after separation from their common ancestor.

## Introduction

Mycoplasmas are a distinct class of prokaryotes that are wall-less, free-living and small in size and can pass through bacteriologic filters. They have unusually small genomes with sizes of 0.5–1 Mbp and have nearly the minimal gene set required for metabolism, showing unusual nutritional needs. These abnormal phenotypic characteristics are the major basis for defining these species as Mollicutes. *Mycoplasma* is used as the general name for species in the class Mollicutes. In the traditional method of classification, Mollicutes is the sole class of Tenericutes and is composed of five orders: Acholeplasmatales, Anaeroplasmatales, Entomoplasmatales, Haloplasmatales and Mycoplasmatales, among which the genus *Mycoplasma* of the order Mycoplasmatales contains the largest number of species, which at over 120. One widely accepted hypothesis states that mycoplasmas evolved from the ancestors of gram-positive bacteria by several rounds of genome reduction (Rogers et al., 1985; Sladek, 1986). Structural information from the histidine-containing phosphocarrier protein of *Mycoplasma capricolum* provided solid evidence for the evolutionary divergence of *Mycoplasma* from gram-positive bacteria (Pieper et al., 1995). Based on 16S rRNA phylogenetic studies of mycoplasmas, Woese et al., hypothesized that Mollicutes are not a phylogenetically coherent group in which all species are derived from a common ancestor; instead, they may represent a diverse collection of wall-less microorganisms derived from many different branches of bacteria (Woese et al., 1980). However, this theory has not been proven, and recent studies have still suggested that Mollicutes originated from one common Gram-positive bacterial ancestor or that Mollicutes have a monophyletic origin within Firmicutes (Ciccarelli et al., 2006). However, Woese’s studies presented a new method of Mollicutes classification, which includes five phylogenetic groups: the hominis group, the pneumoniae group, the spiroplasma group, the anaeroplasma group and the asteroleplasma groupare (Weisburg et al., 1989). Recently, a new classification method based on genome sequence data suggested the creation of the new order Mycoplasmoidales ord. nov., containing two new families (Gupta et al., 2018). Currently, the results of phylogenetic analyses of Mollicutes are controversial.

Sequence and structural similarities among ancient protein orthologs have not changed substantially over the past billion years (Konaté et al., 2019). Enolase (EC number: 4.2.1.11), which is an evolutionarily conserved enzyme found in archaea, eubacteria, plants, animals and humans (Antikainen et al., 2007; Kang et al., 2008; Sun et al., 2017), is one of these molecules (Konaté et al., 2019). Acting as a phosphopyruvate hydratase in several steps of glycolysis, enolase is necessary for survival (Wold and Ballou, 1957a; Wold and Ballou, 1957b). In addition to its catalytic function, enolase has many other functions related to different biological processes. Human enolase has been reported to play roles in many diseases, such as cancers (Wang et al., 2020; Yang et al., 2020), autoimmune disorders (Adamus, 2017), infections (Bergmann et al., 2013), and ischemia (Haupt et al., 2016). Enolase acts as a moonlighting protein in various pathogens. Mhp Eno was reported to be a cell surface-localized protein that can interact with host fibronectin (FN), factor H and plasminogen (PLG) (Chen et al., 2019). Enolase from *Mycoplasma pneumoniae* can directly interact with all other glycolytic enzymes and is considered the central enzyme in glycolysis (Dutow et al., 2010). *Mycoplasma bovis* enolase was found to enhance the adherence of *M. bovis* to embryonic bovine lung (EBL) cells via plasminogen (PLG) (Song et al., 2012). Enolase could also bind RNase E degradosomes and participate in nucleic acid metabolism in *Bacillus subtilis* and *Escherichia coli* (Newman et al., 2012; Bruce et al., 2018). However, most of these extra functions are related to the ability of enolase to bind plasminogen, which is a component of the enzyme system involved in degrading fibrin and extracellular matrix (Godier and Hunt, 2013). However, how conserved enolases have evolved to gain such various functions in different species is unknown.

The first enolase structure was determined in the 1990s (Stec and Lebioda, 1990), after which, structures of enolases from various species were resolved (Duquerroy et al., 1995; Hosaka et al., 2003; de A S Navarro et al., 2007; Kang et al., 2008; Lu et al., 2012). All these structures show that enolases share the same overall protein folding with an N-terminal cap domain and a C-terminal TIM barrel-like domain. The catalytic state of the enzyme is related to its conformation, which can be “open” or “closed”, corresponding to states of enzyme activation or deactivation, respectively (Wu et al., 2015). Enolase adopts two oligomeric states, with all eukaryotic enolases being dimers (Stec and Lebioda, 1990; Duquerroy et al., 1995; Kang et al., 2008) and most prokaryotic enolases being octamers (Brown et al., 1998; Hosaka et al., 2003; Lu et al., 2012; Wu et al., 2015). The first solved *mycoplasma* enolase structure, the octamer Mhp Eno, shows S3/H1, H6/S6, H7/H8, and H13 loop regions that are much longer than those of other enolases, which is a feature not been seen in other resolved enolase structures (Chen et al., 2019).

In recent decades, only a few strictly conserved genes, such as the elongation factor Tu and ATP-synthase beta-subunit, have been used to deduce the phylogenetic relationships of bacteria (Ludwig et al., 1993; Baldauf et al., 1996). Functional conservation is known to limit divergent protein evolution (Konaté et al., 2019). In recent years, comparative structural studies on specific proteins have provided clues for research of the evolutionary relationships of species (Pieper et al., 1995) or specific systems (Prada et al., 2006). In this study, we focused on enolases, to seek clues about *Mycoplasma* evolution by structural comparisons, sequence alignment, phylogenetic and other analyses.

## Materials and Methods

### Timetree Calculation

The evolutionary timeline for Mycoplasmataceae was calculated by TimeTree in timeline mode using the website http://www.timetree.org/. Divergence times for *M. pneumoniae* and *Mycoplasma hyopneumoniae* and for *M. hyopneumoniae* and *M. bovis* were located by using node time mode on the same server.

### Sequence Alignment

Enolase sequence alignment was performed by using Clustal Omega (https://www.ebi.ac.uk/Tools/msa/clustalo/) with the default settings. There were two sources for *mycoplasma* enolase sequences. First, partial sequences were from the complete annotated genome sequences (Supplementary Dataset S5). The rests were from the non-redundant protein sequences database. For the same species, only one strain was used. The incomplete sequences and controversial sequences were discarded. Finally seventy-three *mycoplasma* enolase sequences were collected. To maintain consistency with the structural data, nonmycoplasma enolases were selected preferentially when their structure was known. Considering that mycoplasmas were the main body of the alignment, only representative species were selected from among spiroplasmas, archaeas, eukaryotes, and cell-walled bacterias.

### Protein Expression and Purification

Genes coding Mb Eno (NCBI accession number: WP_013456550) and Mp Eno (NCBI accession number: WP_010874963) were optimized according to *E. coli* codon usage and synthesized by GenScript Biotech Corp. (Nanjing). Then, the two genes were inserted into the pET21a vector and transformed into the BL21(DE3) *E. coli* strain. Protein expression and purification were carried out according to the previous methods (Chen et al., 2019).

### Crystallization and Structural Analyses

Mb Eno and Mp Eno were concentrated and diluted to 6 and 12 mg/ml for crystal screening. The crystals were screened by using the sitting drop vapor diffusion method at 291 K. Mp Eno crystals were grown in 0.2 M sodium citrate tribasic dihydrate, 0.1 M Tris-hydrochloride and 30% v/v polyethylene glycol 400 at pH 8.5 after 7 days. Mb Eno crystals were grown in 0.14 M calcium chloride dihydrate, 0.07 M sodium acetate trihydrate, 14% v/v 2-propanol and 30% glycerol at pH 4.6 after 1 week. The Mp Eno and Mb Eno crystals were diffracted at 1.8 and 1.7 Å, respectively, on beamline BL19U1 at the Shanghai Synchrotron Radiation Facility (SSRF). X-ray diffraction data were merged, integrated and scaled by using HKL3000 software. The structures of Mp Eno and Mb Eno were solved by molecular replacement using the enolase structure of Mhp Eno (Protein Data Bank [PDB] ID: 3j6i) as a reference model with Phaser in the CCP4 program suite (Hough and Wilson, 2018). REFMAC5 (Kovalevskiy et al., 2018) was used for initial restrained rigid-body refinement. COOT (Emsley and Cowtan, 2004) was used for manual model building. Further refinement was performed with Phenix (DiMaio et al., 2013). Finally, the stereochemical quality of the final models was further evaluated with the program PROCHECK. Structural analysis was performed by using the CCP4 program and PyMOL software. Structural similarity assessment and the generation of heat maps were carried out with the Dali server (http://ekhidna2.biocenter.helsinki.fi/dali/). The oligomerization interfaces were analysed by PISA server (https://www.ebi.ac.uk/pdbe/pisa/).

### Negative Staining

Mb Eno and Mp Eno were diluted with PBS to a concentration of 0.01 mg/ml. After glow-discharge treatment, 5 μl of each sample was added to the front side of the copper grid. After standing for 60 s, the copper grid was dried with filter paper. Five microliters of 2% uranyl acetate was immediately added to the copper grid for staining and then dried with filter paper. The staining step was repeated once, after which the grid was dried naturally. The samples were observed with an FEI Tecnai Spirit electron microscope.

### Evolutionary Trees

The enolase sequences used to construct the evolutionary tree were the same as it used in sequence alignment. The evolutionary tree was generated by MEGA 10 (Kumar et al., 2018). Maximum likelihood was used as the statistical method. After protein sequence alignment was carried out, the amino acid substitution models were searched using the aligned sequences. LG mode was used for the substitution model. The gamma distribution was used to calculate rates among sites. Partial deletion was chosen for gap/missing data treatment. All other parameters were set to the default values. To construct the 16S rRNA tree, GTR + G + I mode was used for the substitution model (Kumar et al., 2018). The neighbor-joining tree method was used for statistical test.

### Analysis of Horizontal Gene Transfer

Two hundred eighty-one complete *mycoplasma* genomes (sixty-three species) were downloaded form GeneBank (Supplementary Dataset S5). Genomes without annotations, genomes with mistaken annotations, and genomes with incomplete data were discarded. HGTree database (http://hgtree.snu.ac.kr/) and DarkHorse HGT Candidate Resource (http://darkhorse.ucsd.edu/) were used for the detection of lateral gene transfer and the identification of phylogenetically atypical proteins on a genome-wide basis (Podell and Gaasterland, 2007; Jeong et al., 2016). The ACLAME database (http://aclame.ulb.ac.be), ICEberg 2.0 (http://db-mml.sjtu.edu.cn/ICEberg/) and PHASTER (http://phaster.ca/) were applied to search for integrating conjugative elements, mobile genetic elements, *cis*-mobilizable elements and phage and prophage sequences (Leplae et al., 2010; Arndt et al., 2016; Liu et al., 2019).

### Surface Plasmon Resonance Analysis

SPR was performed at 25°C with a CM5 sensor chip in a BIAcore X100-Plus (GE Healthcare). Purified swine PLG and FN (Sigma) were separately diluted to 50 μg/ml and immobilized on the chip with a resonance unit (RU) of approximately 2000 by using an amine coupling kit (Biacore AB). Binding kinetics were analyzed by using HBS-EP buffer consisting of 10 mM HEPES, 150 mM NaCl, 3 mM EDTA, and 0.05% (v/v) P20 (a surfactant) (Biacore AB). Analytes (Mp Eno and Mb Eno) at a series of increasing concentrations (0–4,000 nmol/L) were used in the experiment. The dissociation phase was monitored for 1,000 s by allowing the proteins to flow over the chip at a flow rate of 30 μl/min. Kinetic model equation $K_{\text{D}}\;=\;\;\frac{k_{d}}{k_{a}}$ (*K*_D_ is equilibrium dissociation constant, *k*_a_ is association rate constant, *k*_d_ is dissociation rate constant) was used to calculate affinity values (Day et al., 2002).

### Far-Western Blot Analysis

Far-WB analysis was carried out according to a previous procedure (Chen et al., 2019). In brief, approximately 20 μg of Mhp Eno, Mp Eno and Mb Eno was transferred to a PVDF membrane. BSA was used instead of *mycoplasma* enolases as a negative control. After blocking with skimmed milk, the membrane was incubated with 5 μg/ml plasminogen or fibronectin (Roche). Then anti-plasminogen or anti-fibronectin antibody (Abcam; 1 μg/ml) was used to interact with the membrane as the primary antibody. Finally, horseradish peroxidase (HRP)-conjugated anti-IgG (Boster; 1:5,000 dilution) and electrochemiluminescence kits (Thermo Scientific) were used to detect proteins in the membrane.

## Results

### *Mycoplasma* Enolases are Different From Other Enolases in their Primary Structure Pattern

**FIGURE 1:**
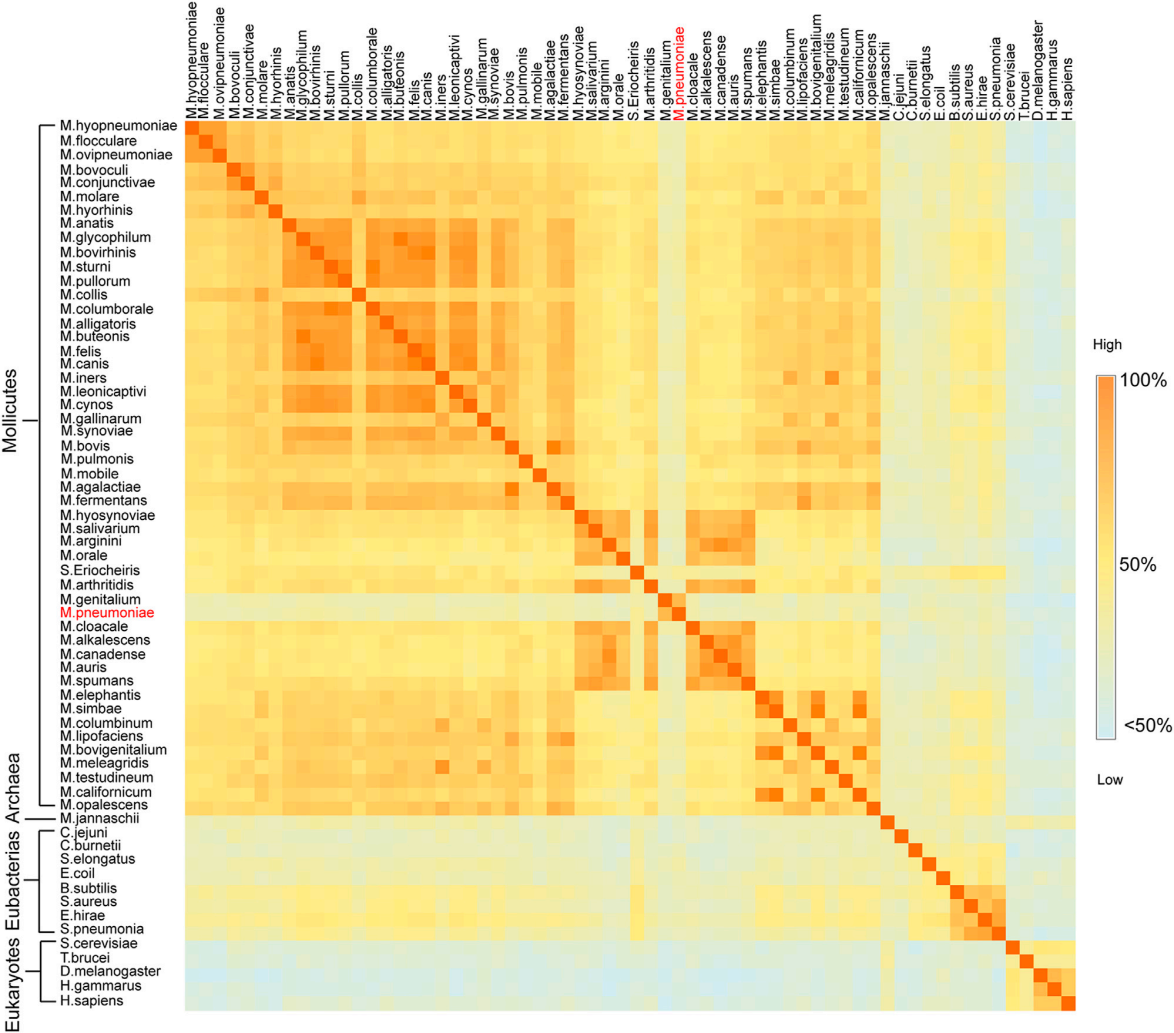
Sequence similarity matrix between two enolases from different species. Heat map of the sequence identity matrix between two enolases from different species. The species names for every row and column are noted. The block shows the sequence identity between the enolases of the two intersecting species. The degree of sequence similarity is colored from dark orange to light blue.

Enolases from different species in a nonredundant protein sequence database were aligned, and *mycoplasma* enolases did not show good sequence similarity with each other. Takeing *M. pneumoniae* enolase (Mp Eno) and *Mycoplasma genitalium* enolase (Mg Eno) as example, the similarity between them was only 77.85% (Figure 1, Supplementary Figure S1 and Supplementary Dataset S1). According to the percent idendity matrix of mammalian enolases, *Streptococcus* enolase and Enterobacteriaceae enolase, the normal enolase similarity between two species with close evolutionary relationships normal is over 90% (Supplementary Figures S2–S4 and Supplementary Datasets S2–S4). For example, the similarity between enolases from *Streptococcus pneumoniae* and *Streptococcus pyogenes* is 93%. Even fly enolase has a percent identity of 78.24% with lobster enolase, although flies and lobsters are only in the same phylum, Arthropoda. Even the similarity between enolases from *Streptococcus pneumoniae* and *Enterococcus hirae* is 83.06%, and these bacteria are members of the same Lactobacillales order but belong to different families (Figure 1, Supplementary Figure S1 and Supplementary Dataset S1). Overall, the identity between any two *mycoplasma* enolases was seldom higher than 90%. The lower similarities between Mp Eno or Mg Eno and other *mycoplasma* enolases were more obvious (Figure 1, Supplementary Figure S1 and Supplementary Dataset S1).

**FIGURE 2:**
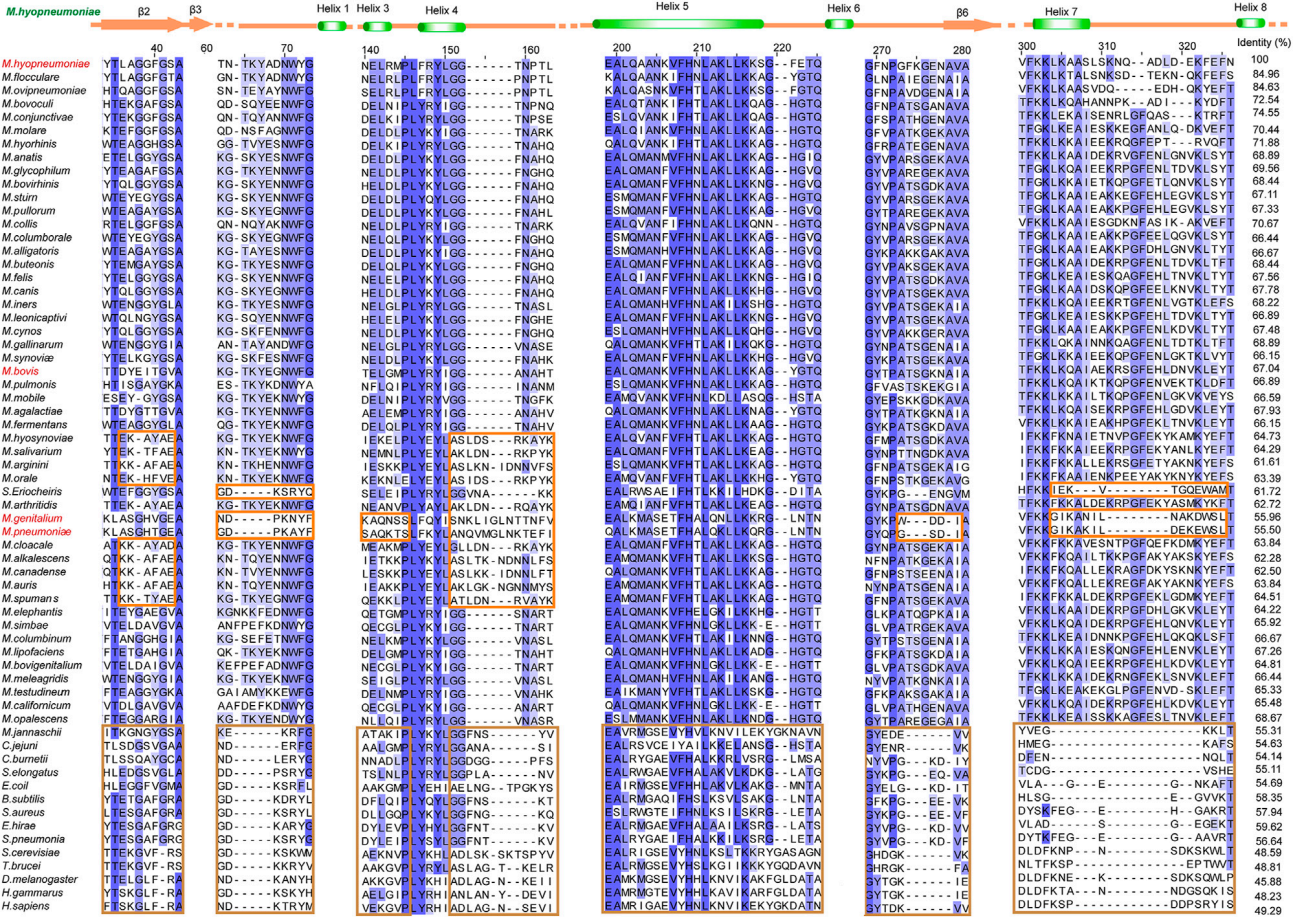
Different sequence patterns among different enolases. Partial sequence alignment of enolases from different species. The full sequence identities are shown at the end of each sequence. The secondary structure of Mhp Eno is shown above the alignment. Different sequence patterns are highlighted with orange and brown rectangles for Mollicutes and non-Mollicutes, respectively. For sequence accession numbers, please refer to supplementary materials.

In our previous studies, the S3/H1, H6/S6, H7/H8, and H13 regions were found to be unique features of Mhp Eno in terms of both sequence and structure (Chen et al., 2019). Here, we confirmed that all *mycoplasma* enolases have a sequence pattern that obviously differs from those of other (archaea, cell-walled bacteria and eukaryotic) enolases, especially in the above-mentioned S3/H1, H6/S6, and H7/H8 regions and H5/H6 region (Figure 2). This sequence pattern also differs among enolases from different *Mycoplasma* species. In the S2/S3 loop, four obviously different sequences were found in total of seventy-six mycoplasma-related enolases (three spiroplasma enolases). The S3/H1 region (containing inserted TKYE) was found to be a specific feature of Mhp Eno in our previous studies. However, we found that fifty-eight *mycoplasma* enolases had this region, but the enolases of *S. eriocheiris*, *M. pneumoniae* and *M. genitalium* lacked this region, showing the same sequence pattern contained in enolases from cell-walled bacteria and eukaryotes (Figure 2). Fifty-three *mycoplasma* enolases contained a deletion between site 150 and site 160 in the H4/S4 loop region; however, only twenty-three *mycoplasma* enolases had an insertion in this region (Figure 2). Other minor differences in the H3/H4 H5/H6, H6/S6, H7/H8 and H11/S10 regions were found among the different *mycoplasma* enolases (Figure 2). Compared to *mycoplasma* enolases, enolases from other organisms in the same phylum or domain share a relatively conserved sequence pattern (Figure 2). These results indicate that *mycoplasma* enolases adopted their own sequence patterns but that these patterns are not strictly conserved.

### Dimeric and Octameric Isoforms are Both Found in Mycoplasma Enolases

**FIGURE 3:**
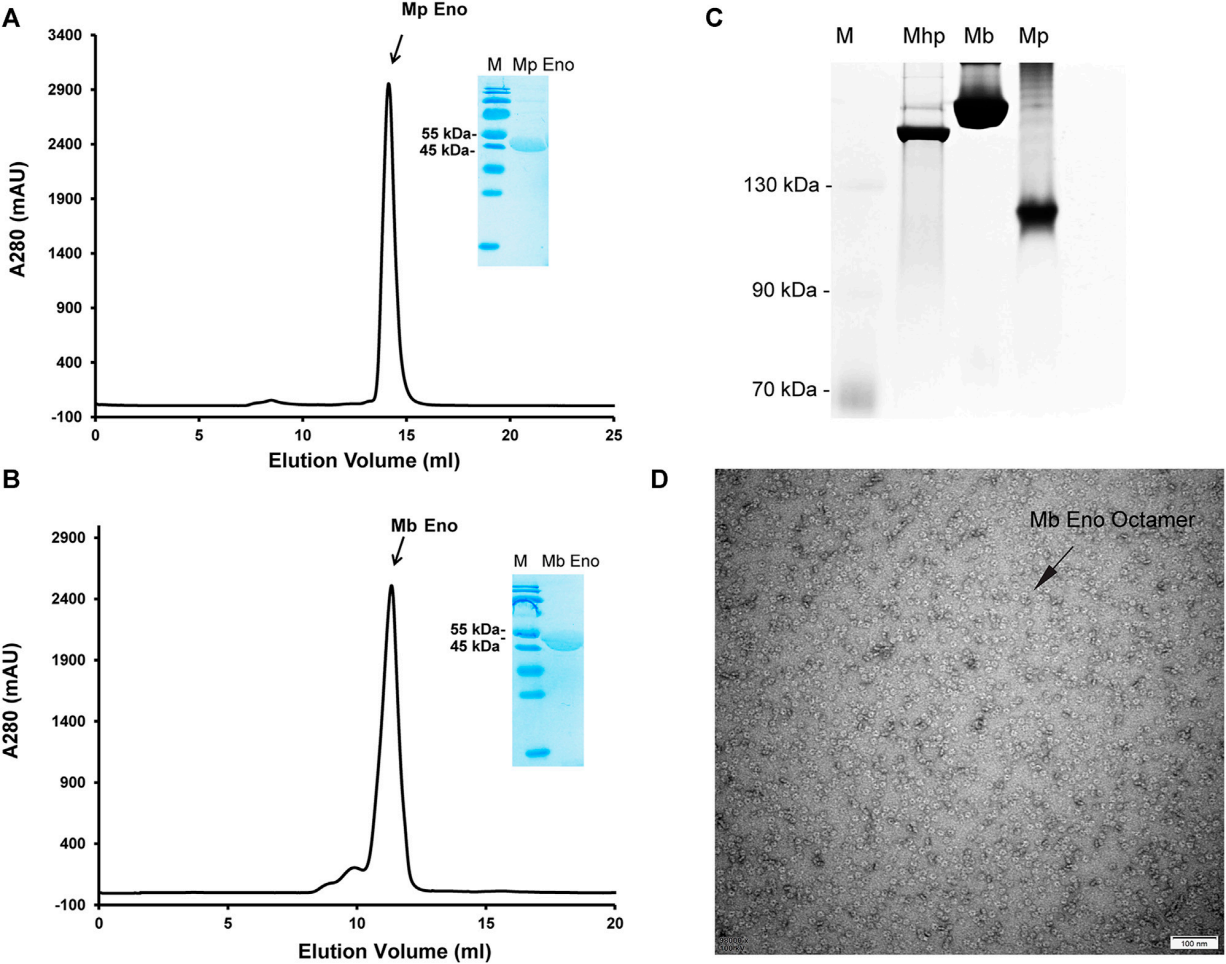
Mp Eno and Mb Eno oligomeric conformations. **(A,B)** Gel filtration of Mp Eno **(A)** and Mb Eno **(B)**. Mp Eno and Mb Eno purities were checked by SDS-PAGE and are shown in the inset images. **(C)** Native PAGE analysis of Mhp Eno, Mb Eno and Mp Eno. The protein marker sizes are indicated to the right of the picture. **(D)** Negative staining of Mb Eno; the scale is indicated.

**FIGURE 4:**
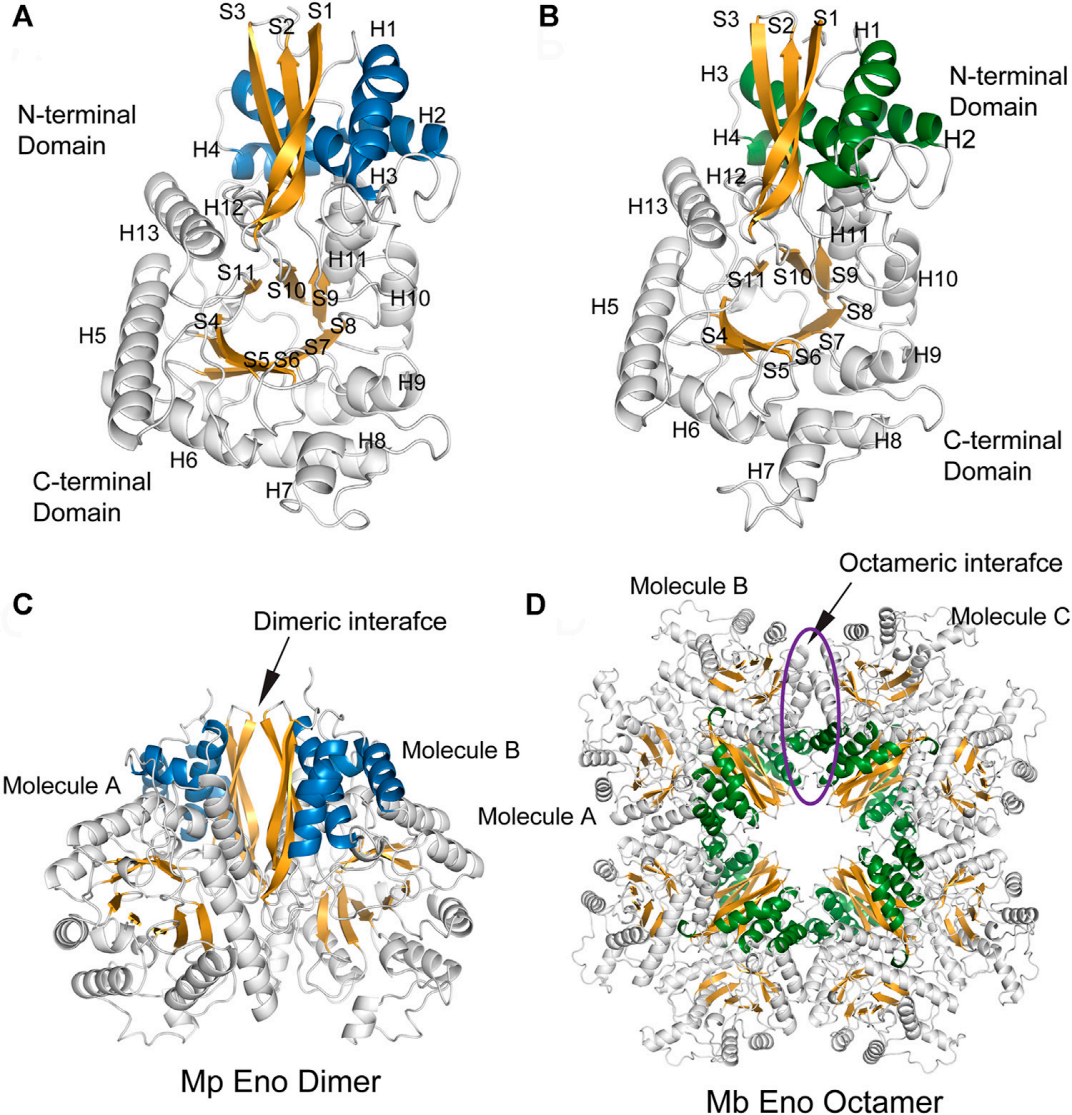
The overall structures of Mp Eno and Mb Eno. **(A,B)** Structures of one molecule each of Mp Eno **(A)** and Mb Eno **(B)**. β-Strands and α-helices are sequentially numbered; “S” indicates a β-strand, and “H” indicates an α-helix. The β-strands are shown in orange. The α-helices of the N-terminal domains are shown in blue and green, in Mp Eno and Mb Eno, respectively. **(C,D)** Overall structures of Mp Eno **(C)** and Mb Eno **(D)**. **(C)** Mp Eno is a dimer. **(D)** Mb Eno is an octamer.

The enolases of most prokaryotes were found to be octamers. However, dimeric enolases have also been observed in some species (Kühnel and Luisi, 2001; Franklin et al., 2015). In our studies, enolase from *M. pneumoniae* was found to be a dimer, and that from *M. bovis* was shown to be an octamer. These results were confirmed by multiple methods. First, denatured Mp Eno and Mb Eno showed the same monomeric molecular weight of 50 kDa on an SDS-PAGE gel (Figures 3A,B). For native PAGE analysis, octameric Mhp Eno was used as a standard molecule. The Mp Eno band ran at approximately 100 kDa, which is between 90 and 130 kDa. The Mb Eno band was above 130 kDa, which is near the band corresponding to Mhp Eno (Figure 3C). This means that soluble Mp Eno and Mb Eno were dimers and octamers, respectively. Second, during size-exclusion chromatography, Mp Eno eluted at approximately 14 ml, corresponding to a molecular weight of 100 kDa, which indicates the dimeric isoform. Mb Eno eluted at approximately 11 ml, corresponding to a molecular weight of 400 kDa, which means that Mb Eno is an octamer. Third, negative stain electron microscopy of the proteins was used to directly image the two molecules. According to the EM images, Mb Eno appeared as a flower-like ring composed of eight small subunits, indicating an octamer. However, Mp Eno was too small to observe its appearance (data not shown) (Figure 3D). Finally, in the resolved structures, Mp Eno is clearly a dimer, and Mb Eno is an octamer (Figure 4). The contacting interfaces for the oligomerization of Mp Eno and Mb Eno were examined. The detail information was listed in Table 1. There is only a dimeric interface that holds the two monomers together in Mp Eno. Mp Eno dimeric interface has 22 hydrogen bonds and buries a total of 1717 Å^2^ surface area. There are 54 amino acids from one molecule and 53 amino acids from another molecule to form the Mb Eno interface. For Mb Eno, however, there are two types of oligomerization interfaces: dimeric interface and octameric interface. The octameric interface fastens the neighboring heart-like dimers to from a ring-like octamer. The dimeric interface of Mb Eno was composed of 49 residues from one monomer and 51 residues from another monomer. There are 34 hydrogen bonds, 12 salt bridges and lots of Van der Waals forces to form an interface area of about 1,856.4 Å^2^. The octameric interface of Mb Eno is on the opposite side of that of the dimer, forming a surface area of about 1,279.9 Å^2^. A total of 67 residues from the two neighboring enolase monomers were involved in this interface. There are 21 hydrogen bonds and 2 salt bridges contributing to the formation of octameric interface (Table 1). In our studies, the oligomerizations of Mp Eno and Mb Eno seem to be stable in different buffers (Tris−HCl buffer and PBS) and different concentrations (>6000 μg/ml and 10 μg/ml). Phenomenon of coexist of monomers, dimers, and octamers in solution found in enolase from *Trichomonas vaginalis* was not detected in Mp Eno and Mb Eno (Mirasol-Meléndez et al., 2018). All these results clearly demonstrate that Mb Eno exists as an octamer and that Mp Eno exists as a dimer. In most cases, enolases from two evolutionarily related species share the same oligomerization state. For example, enolases from both *Streptococcus pneumoniae* (Ehinger et al., 2004) and *Streptococcus suis* (Lu et al., 2012) are octamers. Therefore, it is somewhat unusual that different isoforms are found in *mycoplasma* enolases.

**TABLE 1:** The interactions and amino acids between two chains for the formation of different types of enolase interfaces.

**Enolase**	**MP dimeric interface**	
Interaction	Molecule A	Molecule B
Residues	F16, A17, Y18, Q19, V20, **F21**, D22, **S23**, R24, **G25**, F26, P27, **E42**, M44, **K65**, A66, **Y67**, F68, **D171**, H190, L193, **K194**, **S197**, **E198**, F200, **H201**, A202, Q204, K205, K208, **N213**, T214, N215, **K216**, G217, D218, A223, A242, A243, T395, **E396**, T398, M417, S418, **R419**, **S420**, **E421**, I423, A424, Y426, **N427**, L430, **Q431**, L43	F16, A17, Y18, Q19, V20, **F21**, D22, **S23**, R24, **G25**, F26, P27, V32, **E42**, M44, **K65**, A66, **Y67**, F68, K135, D171, H190, L193, **K194**, **S197**, **E198**, F200, H201, Q204, L212, **N213**, T214, N215, **K216**, G217, D218, A223, T395, **E396**, T398, M417, S418, **R419**, **S420**, **E421**, I423, A424, Y426, **N427**, L430, **Q431**, L434, E435
Interface area (Å^2^): 1,765.0		
**Enolase**	**MB dimeric interface**	
Interaction	Molecule A	Molecule B
Residues	R6, Q8, ***R10***, ***E11***, I12, **L13**, D14, **S15**, R16, **G17**, **N18**, P19, Q22, E24, G60, N61, W62, **F63**, M69, S161, ***R180***, L183, Q184, **N187**, **K188**, F190, **H191**, N192, K195, G203, **Q205**, **V206**, **G207**, **A213,** T397, **E398**, T400, M419, S420, **R421**, **T422**, **D423**, I425, A426, Y428, **N429**, L432, V433, ***E436***	R6, **Q8**, ***R10***, ***E11***, **I12**, **L13**, D14, **S15**, R16, **G17**, N18, P19, Q22, E24, **G60**, N61, W62, **F63**, M69, S161, ***R180***, L183, Q184, **N187**, **K188**, F190, **H191**, N192, K195, G203, T204, **Q205**, **V206**, **G207**, **A213**, T397, **E398**, T400, M419, S420, **R421**, **T422**, **D423**, I425, A426, Y428, **N429**, L432, V433, ***E436,*** E437
Interface area (Å^2^): 1,856.4		
**Enolase**	**MB octameric interafce**	
Interaction	Molecule B	Molecule C
Residues	**F89**, D90, Q91, **R92**, A93, ***K96***, L130, M132, **R136**, **Y137**, **I138**, G139, G140, **A141**, **N142**, H144, L371, M375, **D376**, N379, Q382, **K383**, A384, **N385**, **F409**, **N410**, L438, E440, **Q441**, E443, Y451, K454	**F89**, D90, Q91, **R92**, A 93, ***K96***, L130, M132, **R136**, **Y137**, **I138**, G139, G140, **A141**, **N142**, H144, E354, K356, L371, M375, **D376**, N379, Q382, **K383**, A384, N385, F409, **N410**, L438, ***E440***, **Q441**, S442, E443, E445, Y451
Interface area (Å^2^): 1,279.9		

Bold: hydrogen bond; italics: salt bridge; regular: Van der Waals force.

### The Unusual Structures of Mp Eno and Mb Eno

The structures of enolase Mp Eno and Mb Eno crystals were determined at 1.8 Å and 1.7 Å respectively (Table 2). As enolase is a relatively conserved enzyme, Mb Eno and Mp Eno have overall structures very similar to those of other enolases (Figures 4A,B). Dimeric Mp Eno is composed of two enolase monomers with a heart-like or butterfly like shape, similar to the shapes of other dimeric enolases (Figure 4C). Octameric Mb Eno consists of four dimers of enolase monomers, forming a disc-like shape with a center tunnel (Figure 4D). Both Mb and Mp enolase monomers are composed of an N-terminal cap domain and a C-terminal TIM barrel domain. The C-terminal domain of Mb Eno and Mp Eno shows a topology of β2α2βα2(βα)5, which differs from the traditional β2α2(βα)6 topology of other enolases (Figures 4A,B).

**TABLE 2:** Data collection and refinement statistics.

	Mp Eno	Mb Eno
Data collection		
Beamline	SSRF BL19U1	SSRF BL19U1
Space group	*P*1 21 1	*I*4
Cell dimensions		
*a*, *b*, *c* (Å)	76.074, 106.548, 128.589	142.221, 142.221, 107.422
α, β, γ (°)	90.00, 103.15, 90.00	90.00, 90.00, 90.00
Wavelength (Å)	0.97853	0.97853
Resolution (Å)	19.84-1.8 (1.9-1.8)	19.96-1.7 (1.79-1.7)
Total no. of reflections	368478 (36808)	115149 (9944)
*R*_merge_ (%)	0.105	0.071
*Ι*/σ*Ι*	6.55 (1.33)	2.15
Completeness (%)	98.5 (98.7)	98.1 (98.1)
Redundancy	6.4	11.2
Refinement		
Resolution (Å)^a^	19.84-1.8	19.72-1.7
No. reflections	181,623	114,827
R, R_*free*_	0.189 (0.223)	0.158 (0.182)
No. atoms	14,600	7,242
Protein	13,715	6851
Water	837	392
Average *B*-factors (Å^2^)	29.40	32.98
Protein	29.08	32.83
Water	34.21	35.62
Rmsd values		
Bond lengths (Å)	0.007	0.015
Bond angles (°)	0.83	1.30
Ramachandran plot (%)		
Total favored	98.04%	97.75%
Total allowed	1.85%	1.91%
Outliers	1.11%	0.96%

a The values in parentheses are for the highest-resolution shell.

Rmerge = P | I - hIi| /PI, where I is the integrated intensity of a given reflection. R = P || Fobs | – | Fcalc | |/Phkl | Fobs |. Rfree was calculated using 5% of the data omitted from the refinement. I /σI = average (I/σI).

**FIGURE 5:**
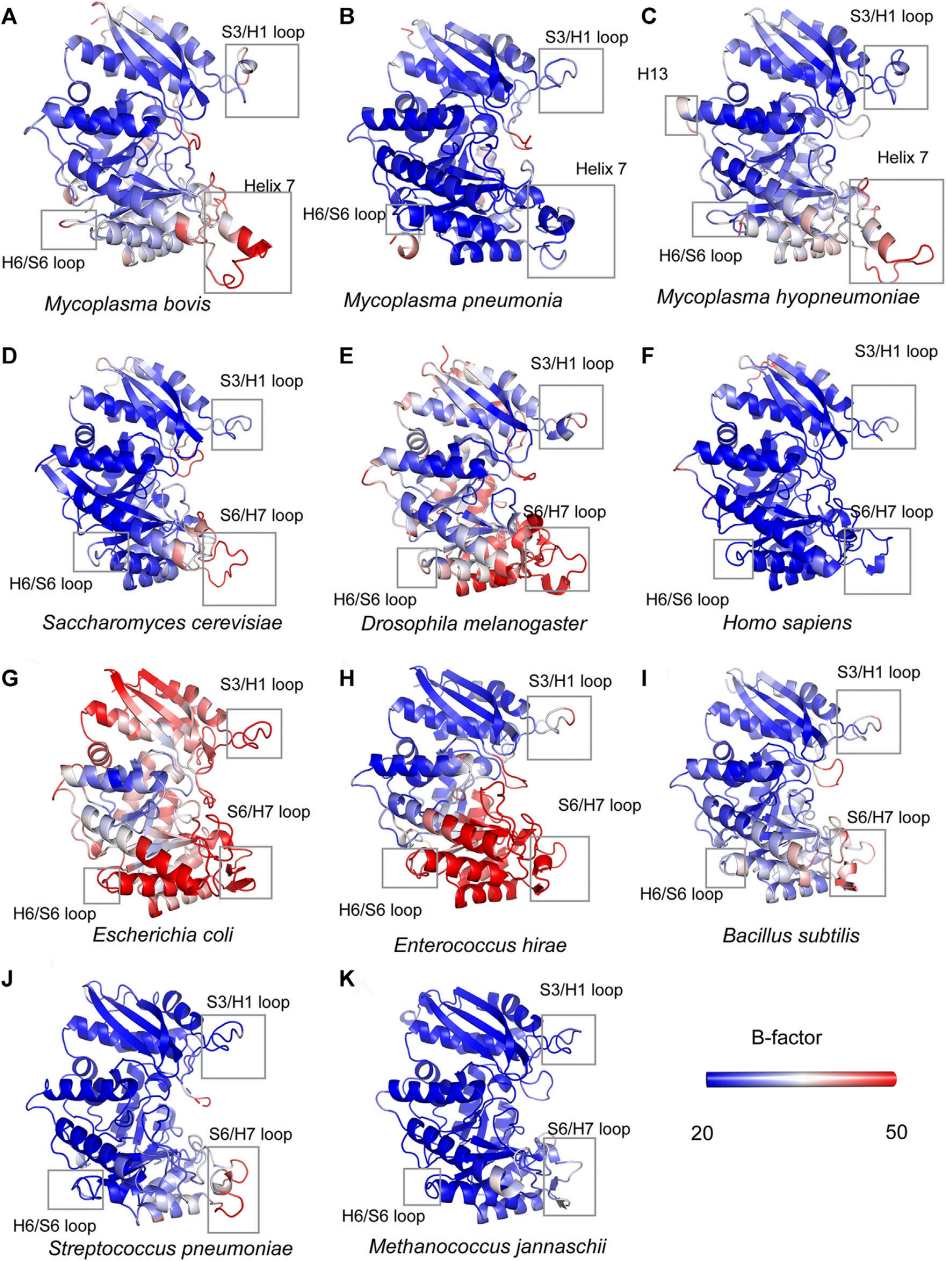
Structural comparisons of *mycoplasma* enolases and other enolases. All the enolases are shown in cartoon mode and in the color of blue-white-red spectrum according to crystallographic B factors. The color bars show the B-factor scales for the corresponding forms. Species names are below corresponding enolase structure modes. Helix 7, H13, H6/S6 loop, S6/H7 loop regions are marked by rectangles. The accession numbers for enolases are the same as used in Supplementary Table S2.

A detailed structural comparison between *mycoplasma* enolase and other determined enolase structures in the PDB database was carried out. First, the structural superimposition of enolases from different species was performed (Supplementary Figure S5). The overall folds of all enolases overlapped well. As indicated by the sequence alignment, notable differences were still found in the S3/H1, H6/S6, H7/H8, and H13 regions (Figure 5 and Supplementary Figure S5). Insertions in these regions were found in *mycoplasma* enolases. This difference manifests as differences in the length of the loop or the insertion or deletion of a helix. However, amino acid mutations were not reflected in the structures. For example, although obviously different amino acid arrangements were observed in the H5/H6 region (Figure 2), no structural differences were detected (Supplementary Figure S5). Thermal stabilities of different enolase structures were also checked (Figure 5). It is clear that S3/H1 loop and H6/S6 loop regions studied here are seems to be stable, while most of them have a low or moderate b-factor value. S6/H7 loop regions or Helix 7 regions have relative high b-factors in their overall structures. However, the values of Helix 7 are still acceptable in *mycoplasma* enolase structures (Figure 5).

To quantify the structural similarity among enolases from different species, “all-to-all” structural comparison was carried out (Supplementary Figure S6). According to the heat map, species with close evolutionary relationships clearly have high structural similarity and are clustered together (Supplementary Figure S6). However, similar to the low identity among *mycoplasma* enolase sequences, structures of *mycoplasma* enolase also did not show good similarity with each other. Both Mb Eno and Mp Eno showed the best structural similarity with *E. hirae* enolase with a Z-score of 61.7. Furthermore, both Mb Eno and Mp Eno showed the second-best similarity with enolase from *B. subtilis*, with a Z-score of 61.4. The Z-score for Mp Eno and Mhp Eno was only 58.5, and that for Mp Eno and Mhp Eno was only 60.9. Mhp Eno is the most structurally similar to the enolases of *B. subtilis*, *S. aureus* and *E. hirae*, with Z-scores of 62.7, 62.2 and 61.5, respectively. The Z-score for Mb Eno and Mhp Eno was only 61.3 (Supplementary Table S1). These analytical results indicate that the structures of *mycoplasma* enolases also differ from one another.

### Helix 7 is a Feature Specific to Mycoplasmataceae

**FIGURE 6:**
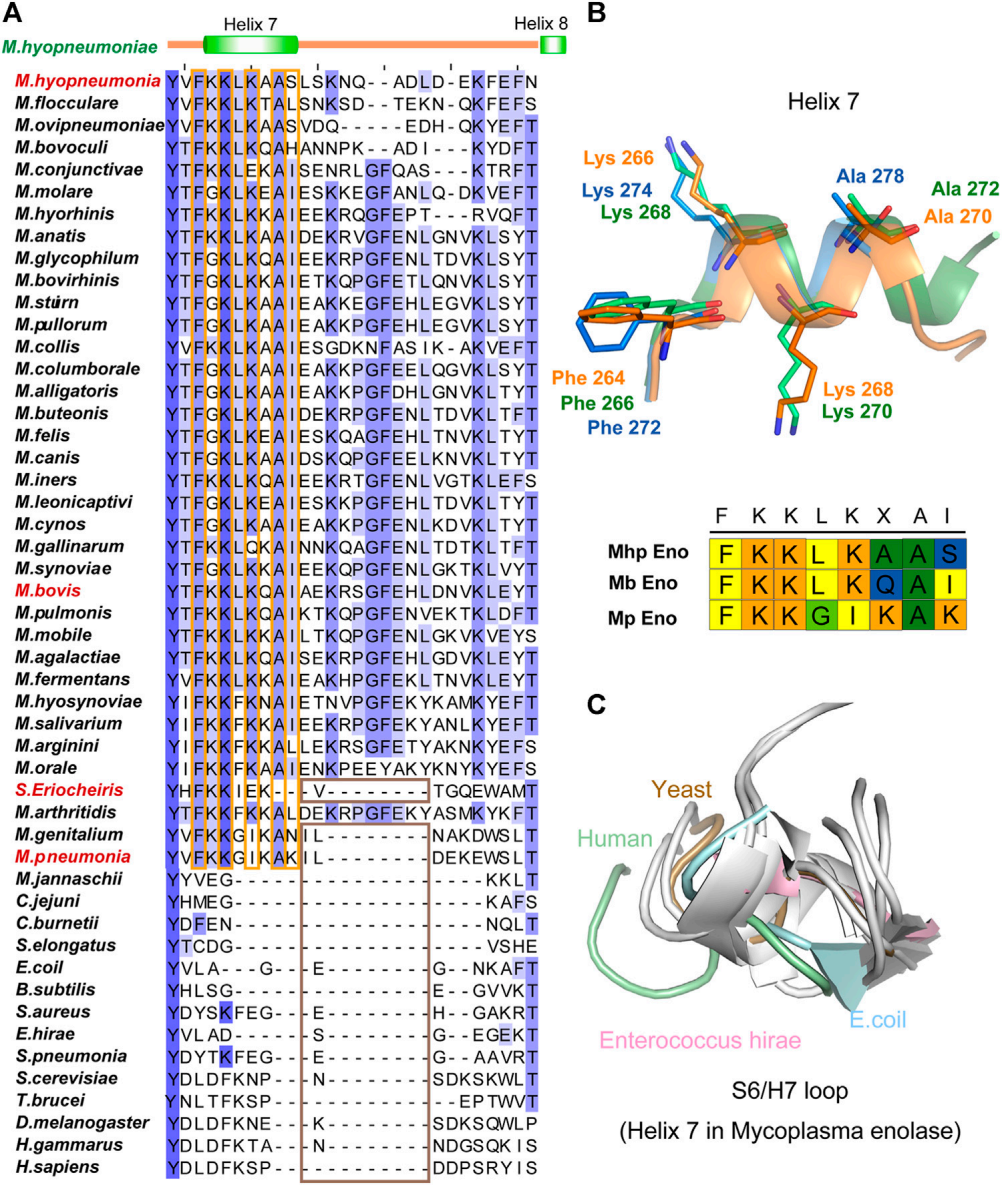
Helix 7 motifs and structures. **(A)** Sequence alignment of the H7/H8 regions of different enolases. For sequence accession numbers, please refer to supplementary materials. The secondary structure of Mhp Eno is indicated above the alignments. The conserved sites in the *mycoplasma* enolase H7 region are highlighted with orange rectangles. The absence of H7 and H8 in enolase structures is highlighted with brown rectangles. **(B)** Structures of helix 7 from three *mycoplasma* enolases. Blue, green and orange indicate Mp Eno, Mb Eno and Mhp Eno, respectively. Conserved sites are indicated and shown stick models. The H7 motifs in Mp enolase, Mb enolase and Mhp enolase are shown under the H7 structures. **(C)** Structural overlap of the S6/H7 loops (corresponding to Helix 7 of *Mycoplasma* enolases) of enolases from non-*Mycoplasma* species. Enolases from *H. sapiens*, *S. cerevisiae*, *E. coli* and *E. hirae* are shown in light green, light blue, brown and pink, respectively, and other enolases are shown in white.

However, unlike other enolases, both Mb Eno and Mp Eno have an extra helix, helix 7 (H7), which was first found in Mhp Eno and shown to be a species-specific loop in Mhp Eno (Figure 5). To determine whether all *mycoplasma* enolases have helix 7, we checked the alignment and found that the amino acids in H7 are relatively conserved among almost all *mycoplasma* enolases (Figure 2, Figure 5 and Figure 6A). Helix 7 is composed of a motif with the sequence of F-K/G-K-L/F-K-X-A-I. Within this motif, the first amino acid, F, and third amino acid, K, are strictly conserved, and the other sites are occasionally substituted by other amino acids. However, all other nonmycoplasma enolases lack this motif (Figure 6). This was further confirmed by structural analysis (Figures 6B,C). Therefore, we confirmed that helix H7 is characteristic to *mycoplasma* enolases in terms of both sequence and structure.

The long loop after helices H7 and before H8 also only exists in *Mycoplasma* species (Figure 2). However, the length of the H7/H8 loop differed among the three *mycoplasma* enolases examined (Supplementary Figure S5B and Figure 6). Mb Eno had the longest H7/H8 loop, which was longer than that of Mhp Eno by 3 additional amino acids. Approximately 85% of the *Mycoplasma* species within Mycoplasmataceae share this long H7/H8 loop sequence.

### Enolases are Conserved Among Different Kingdoms but Divergent in Mycoplasmataceae

**FIGURE 7:**
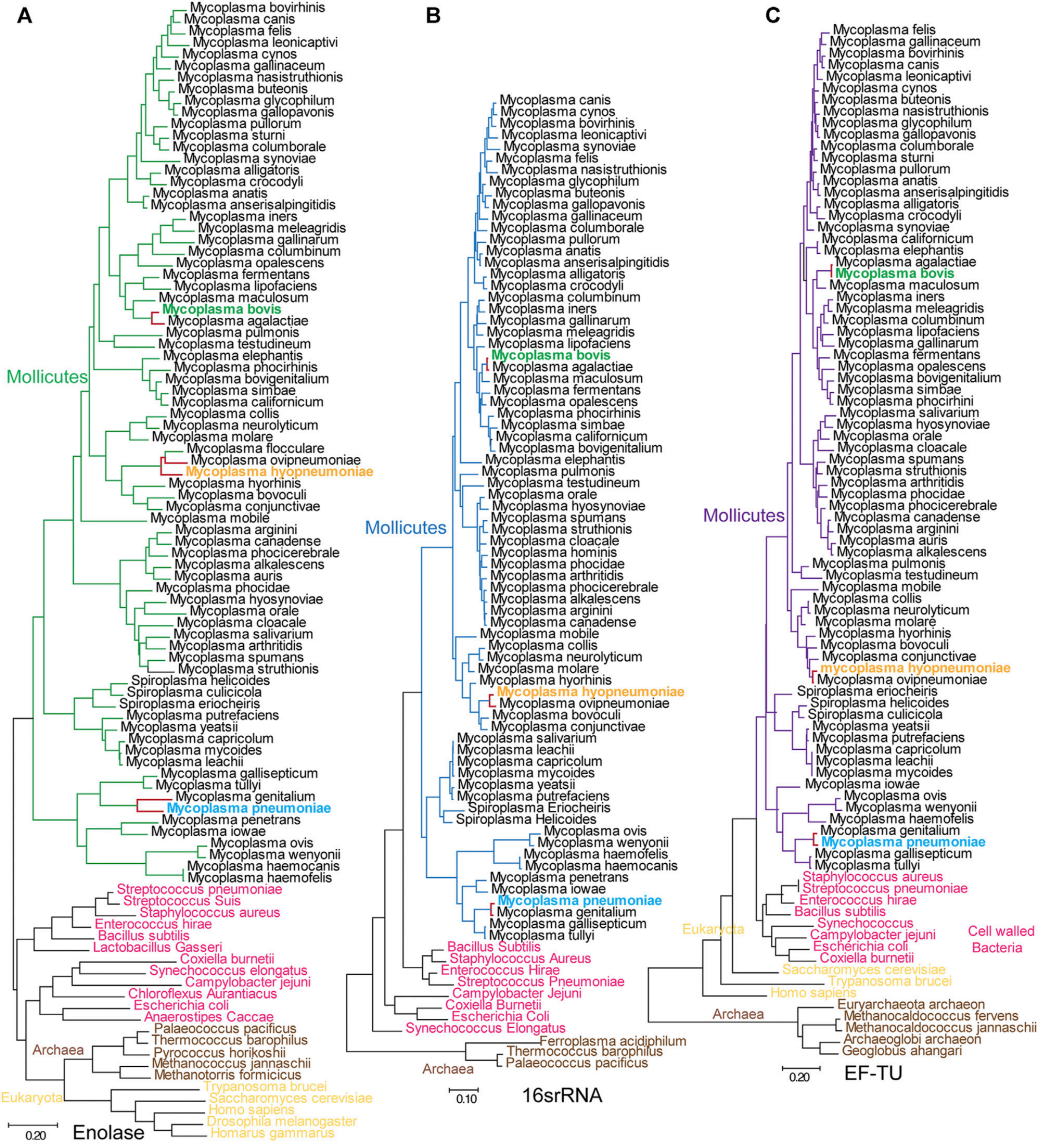
Mycoplasmataceae evolutionary trees generated from enolase, EF-TU and 16S rRNA sequences. **(A–C)** Evolutionary trees generated with enolase, 16S rRNA and EF-TU sequences. Bars indicate distances under the corresponding tree. *Mycoplasma bovis*, *M. pneumoniae* and *M. hyopneumoniae* are shown in green, blue and orange, respectively. The branches for *M. pneumoniae*, *M. genitalium*, *M. hyopneumoni*ae and *M. ovipneumoniae* are red in all three trees to clarify the distance between neighboring species.

To better understand the evolutionary relationship among different enolase proteins, we constructed an evolutionary tree by using enolase protein sequences from different species. We simultaneously constructed a tree by using 16S rRNA sequences from the corresponding species to determine the evolutionary relationship among these specific species (Figure 7). To conduct a parallel comparison, an evolutionary tree was also built using conserved EF-TU proteins (Ludwig et al., 1993) from the corresponding species. It is easily found that the three different evolutionary trees exhibited good reconciliation. Mycoplasmas were grouped into three branches: the pneumoniae group, the hominis group and the spiroplasma group. The evolutionary relationship evidenced by the enolase tree was consistent with that calculated by 16S rRNA tree but was different from the traditional classification. For example, *M. mycoides*, *M. leachii*, *M. capricolum* and several other species are clustered with spiroplasmas other than mycoplasmas. However, as shown by the evolutionary trees, enolase proteins from different kingdoms or phyla have much closer relationships than the overall evolutionary relationships of the species themselves. In other words, the genetic distances between the kingdoms Archaea, Bacteria and Eukarya are much longer than the corresponding distances between their enolase proteins. However, the distances among different *mycoplasma* enolases were much longer than those in the EF-TU and 16S rRNA trees (Figure 7). For example, the distances between *M. hyopneumoniae* and *Mycoplasma ovipneumoniae* were 0.25, 0.1 and 0.04 in the enolase, EF-TU and 16S rRNA trees, respectively. The distances between *M. pneumoniae* and *M. genitalium* were 0.288, 0.031 and 0.017 in the enolase, EF-TU and 16S rRNA trees, respectively. These results indicate that enolases from different *Mycoplasma* species exhibit longer evolutionary distances than the specific species themselves (Figure 7). These results indicate that enolases are conserved among different kingdoms but divergent in Mycoplasmataceae.

Horizontal gene transfer is always a factor of disturbance in evolutionary analyses. However, according to our analysis, it is very unlikely that horizontal gene transfer occured in *Mycoplasma* enolase genes. First, we checked 281 public genomes of mycoplasmas and found that only one enolase gene could be identified in each genome. Second, *mycoplasma* enolase genes are not included in the list of phylogenetically atypical proteins, which are potential candidates for horizontal gene transfer, as calculated by DarkHorse HGT Candidate Resource and HGTree. Third, none of the integrated conjugative elements, mobile genetic elements, *cis*-mobilizable elements, or phage and prophage sequences were detected in *mycoplasma* enolase genes by using ICEberg 2.0, ACLAME or PHASTER. Therefore, we can conclude that *mycoplasma* enolase genes were generated by parent-to-progeny inheritance rather than horizontal gene transfer.

### Divergent Evolution is Specific to Mycoplasmataceae Enolase

To determine whether divergence in enolase exists in all the other clades, enolases from Enterobacteriaceae, *Streptococcus* and Mammalia were randomly selected and used to construct evolutionary trees (Supplementary Figure S7). Enolases from the same class among these Enterobacteriaceae, *Streptococcus* and Mammalia species were closely clustered in the trees. In fact, the distances from one enolase to another in the same class were too short to distinguish. This means that enolases in these species are well conserved or did not undergo much independent evolution (Supplementary Figure S7). However, in contrast, the branches of different species in the *mycoplasma* enolase tree are long enough to be well distinguished (Figure 7A). These results indicate that enolase divergence is specific to Mycoplasmataceae. However, we have not determined whether this finding applies to every class. Currently, this divergence has been found in only Mycoplasmataceae.

### Different Loop Regions Under Different Modes of Evolution

**FIGURE 8:**
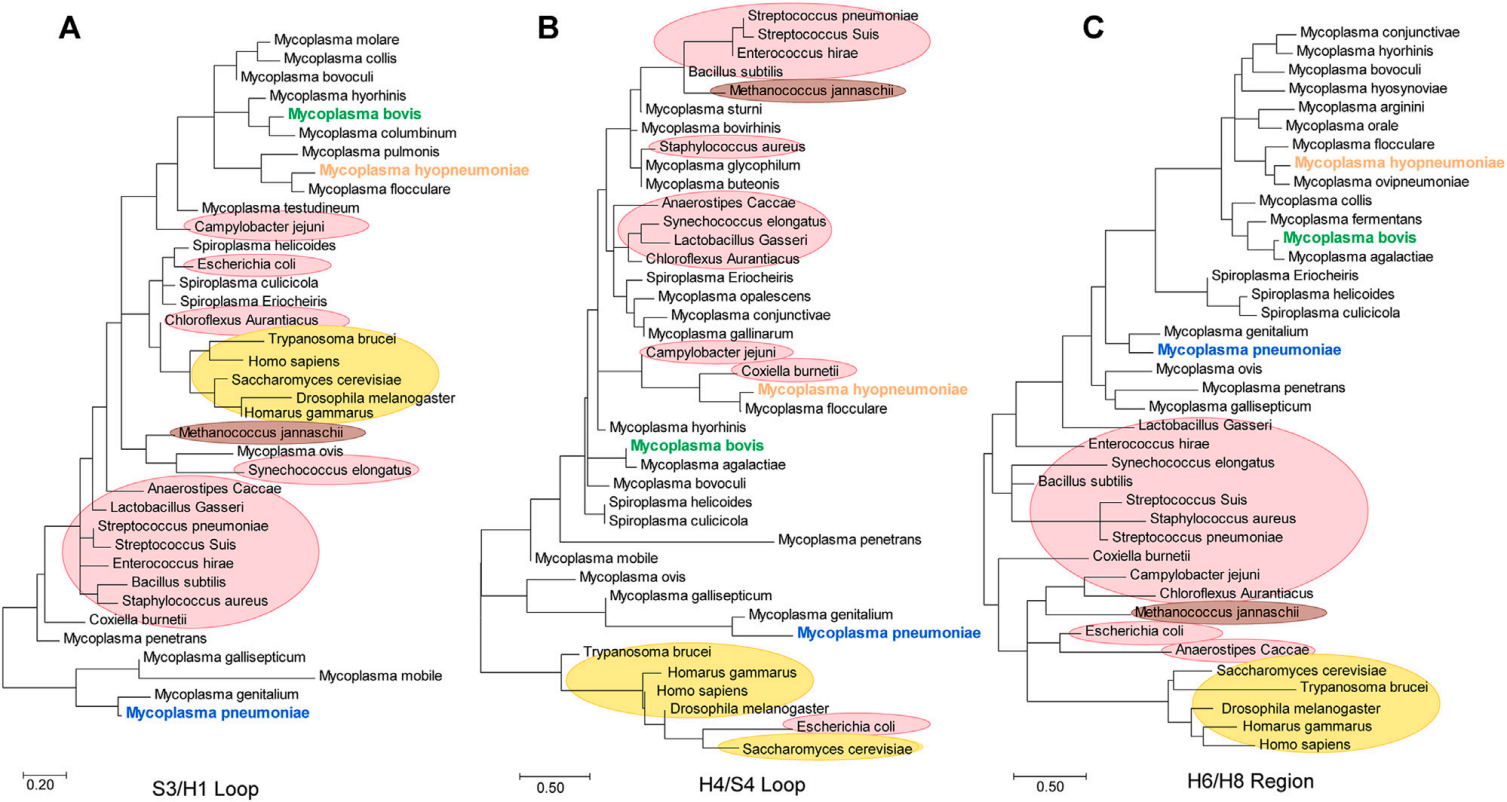
Phylogenetic trees generated from the S3/H1 loop, H4/S4 loop and H6/H8 regions of enolases from Mycoplasmataceae.

Furthermore, we constructed separate evolutionary trees for the S3/H1, H6/H8 (including the H6/S6 loop and H7/H8 loop) and H4/S4 regions (Figure 8). According to the S3/H1 loop tree, the original evolutionary relationship was clearly disrupted. *Mycoplasma ovis* and *Synechococcus elongatus* had the closest relationship. *E. coli* and *C. aurantiacus* were mixed in the Mollicutes clade. *Campylobacter jejuni* showed a closer relationship with eukaryotes than with other species (Figure 8A). The same findings were observed in the H4/S4 loop tree. The H4/S4 loop of *E. coli* enolase had a much closer relationship with those of eukaryotic enolases. Cell-walled bacterial clades were distributed in branches of Mollicutes (Figure 8B). These results indicate that the S3/H1 and H4/S4 regions of *mycoplasma* enolases are hot spots for evolution. However, the H6/H8 region tree and species evolutionary tree were consistent. In the H6/H8 region tree, all Mollicutes species are clustered (Figure 8C). Cell-walled bacteria were also clustered. This indicates that the evolution of this region is quite consistent with the evolution of the corresponding specific species. This is also consistent with the previously observed phenomenon that helix 7 and the H7/H8 loop are almost absolutely conserved in mycoplasmas. This means that the H6/H8 region including helix 7 of *mycoplasma* enolases, may have evolved from a common ancestor. This further confirms the species-specific nature of *mycoplasma* enolases.

### Different Mycoplasma Enolases Show Different Affinities to PLG and FN

Different isoform statuses, structural features and sequence patterns have been observed in different *mycoplasma* enolases. To determine whether they have different functions, we checked their affinity to PLG and FN. Mb Eno, Mp Eno and Mhp Eno are all reported to be important adhesins and can interact with PLG and FN. SPR is widely used for bacteria moonlight enzymes to detect their binding affinities with PLG and FN (Matta et al., 2010; Yu et al., 2018). Therefore, we performed SPR to determine the affinity between PLG/FN and the three enolases. According to the results, the affinities of the three enolases to PLG were different. Mhp Eno had the best value of 62.5 nM. The *K*_*D*_ of Mb Eno was 95.23 nM. The affinity of Mp Eno to PLG was about 300 nM. These results indicate that all three enolases could interact with PLG and showed slight differences from each other (Table 3 and Supplementary Figure S8). For FN, this difference was more significant. Affinity of Mp Eno to FN was too low to be detected. The FN affinity of Mb Eno and Mhp Eno were 485.8 and 74.08 nM respectively, with 10-fold difference (Table 3 and Supplementary Figures S8E–G). These results were also confirmed by far-WB (Supplementary Figure S8) and indicate that *mycoplasma* enolases have similar binding functions, but their binding affinities differ.

**TABLE 3:** Binding affinities of plasminogen/fibronectin with different enolases determined by SPR.

Plasminogen (Plg)			Fibronectin (FN)		
Species	Proteins	*K*_D_ (nM)	Species	Proteins	*K*_D_ (nM)
*M. hyopneumoniae*	Eno	62.50 ± 11.1	*M.hyopneumoniae*	Eno	74.08 ± 6.4
*M. bovis*	Eno	95.2 ± 5.2	*M.bovis*	Eno	485.8 ± 15.3
*M. pneumoniae*	Eno	300.0 ± 8.0	*M.pneumoniae*	Eno	>1,000
*B. anthracis*	GAPDH (Matta et al., 2010)	572	*M.hyopneumoniae*	FBA (Yu et al., 2018)	468

## Discussions

Enolase is an evolutionarily conserved enzyme that has been found in almost all kingdoms, such as Archaea, Bacteria, Plantae and Animalia (Stec and Lebioda, 1990; Duquerroy et al., 1995; Kang et al., 2008; Lu et al., 2012; Chen et al., 2019). However, in our studies, enolases from mycoplasmas exhibited many characteristics that are outside of the norm for enolase. First, in terms of primary structure, *mycoplasma* enolases have their own sequence patterns that can easily separate *mycoplasma* enolases from enolases of other species. Most of these patterns can be reflected in their resolved three dimensional structures. Second, the crystal structures of two *mycoplasma* enolases, Mb Eno and Mp Eno, confirmed that helix 7 is a common structural feature of *mycoplasma* enolases. The motifs of helix 7 were also first recognized herein. Helix 7 endows the structures of *mycoplasma* enolases with a new topology, different from the traditional topology. This motif could also be regarded as a molecular marker specific for *mycoplasma* enolases. To our surprise, neither the sequence identity nor the structural similarity among *mycoplasma* enolases showed high scores. This means that even in the same order or family, *mycoplasma* enolases are not similar to each other. Furthermore, polygenetic analysis of *mycoplasma* enolases showed much longer genetic distances than the distances between the two specific species themselves. This phenomenon has been restrictively observed only in enolases of mycoplasmas. Here, we regard it as divergent evolution that occurred in enolases of mycoplasmas. This may reflect the evolutionary status of mycoplasmas themselves. The extra low sequence/structural similarity between Mp Eno and Mg Eno to other *mycoplasma* enolases may reflect the unique evolution of these two species, which have the highest G + C% among mycoplasmas and probably the longest branch length in the phylogenetic tree. We further independently checked the genetic relationships of the featured loop regions, S3/H1, H4/S4 and H6/H8 (including helix 7) of *mycoplasma* enolases. Different evolutionary modes have been used for the three different regions. However, the confusing genetic relationships shown by S3/H1 and H4/S4 trees indicated a rapid evolutionary pace. The organized tree of H6/H8 regions hints at a conserved evolution mode. Finally, different affinities of the three *mycoplasma* enolases to PLG/FN were also shown. These results suggest that there are functional differences among different enolases. All results presented herein indicate a class of unusual enolases from a group of abnormal bacteria.

Divergent evolution was found only in *mycoplasma* enolases. This may be related to the special evolutionary status and abnormal physiological characteristics of mycoplasmas. This also indicates a rapid evolution of *mycoplasma* enolases or mycoplasmas. First, mycoplasmas arose from Mollicutes and are thought to have undergone two rounds of degenerate evolution (Rogers et al., 1985). The initial event was the formation of the Acholeplasma branch, at which time the genome was reduced to l000 MDa, and the cell wall was lost. Then, several independent reductions in the genome occurred, with each eliminating approximately 500 MDa, resulting in *Mycoplasma* species. Studies have shown that *Mycoplasma* species, especially those with the smallest genomes, have high mutation rates, indicating that they are in a state of rapid evolution (Rogers et al., 1985). Second, enolase is a moonlighting protein in many species. Among *Mycoplasma* species, multifunctionality of enolase has also been found in *M. hyopneumoniae* (Chen et al., 2019), *M. bovi* (Song et al., 2012), *M. pneumoniae* (Dutow et al., 2010), *Mycoplasma fermentans* (Yavlovich et al., 2007) and *Mycoplasma synoviae* (Bao et al., 2014). A system biology approach showed that enolases from eukaryotic organisms may have conserved moonlighting functions, yet certain degrees of difference still exist (Paludo et al., 2015). In our studies, a similar phenomenon was observed in which enolases from three different mycoplasmas could bind PLG and FN, but with different affinities. It is clear that the enolase framework sequence and overall structure are relatively conserved throughout all the species. This may explain the canonical interactions of enolase in glycolytic and nonglycolytic functions. However, conservation was not absolute, and minor differences in sequence patterns were observed among different enolase (Figure 2). Some of these sequence differences were reflected in the 3-D structure, and some were not (Supplementary Figure S5). The primary sequence determines the secondary and 3-D structures. The structure of a protein determines its function. These sequence and structural differences have subtle influences on the functions of enolase. *Mycoplasma* species have reduced genomes that have to perform more functions with fewer proteins (Raymond et al., 2015; Tacchi et al., 2016). Therefore, the rapid evolution of *Mycoplasma* species will increase their chance to respond in a timely manner to circumstance that may threaten their survival. Third, these differences might be the result of coevolution between *Mycoplasma* species and their hosts (Ge and Karzai, 2009). Normally, different *Mycoplasma* species have different specific hosts. Their hosts, span from plants to animals, from arthropods to mammals and from aquatic animals to terrestrial animals (Woese et al., 1980). Plants, insects and animals evolved during different periods (Marin et al., 2017). During long-term coevolution between *Mycoplasma* species and their specific hosts and specific microenvironments, these *Mycoplasma* species have evolved more specialized adaptation mechanisms.

Structures are regarded as constraining factors for the evolution of enzymes or protein families with the same function. In some cases, 3D structural comparison may indicate biologically interesting similarities that cannot be detected by sequence comparison (Rost, 1997). One study showed that the evolution of protein structures was different from the evolution of organisms and adopted a “multiple birth model’’ (Choi and Kim, 2006). These findings may fully or partially explain the differences observed in the evolutionary trees generated using enolase, EF-TU and 16S rRNA. Our studies show that enolase structures from different species are indeed conserved, although some differences in sequence exist. This is consistent with previous structural studies of enolases (Brown et al., 1998; Kang et al., 2008; Wu et al., 2015; Sun et al., 2017; Chen et al., 2019). However, H7 is an exception and has been found only in *mycoplasma* enolases (Figures 2, 4–6). This structural characteristic has not been found in other species and can also be detected by sequence alignment. In terms of both its structure and sequence, H7 can be regarded as a molecular marker to distinguish *mycoplasma* enolases from other enolases. According to the “multiple birth model” theory (Choi and Kim, 2006), enolases are classified as the α-β type, which is the preponderance and “mature” class. The common structural and sequence features of *mycoplasma* enolases indicated that all examined *mycoplasma* enolases shared a common ancestor, which was new born and different from the previous enolase ancestor. The unique helix-7 may be one of the significant features of the newer born common ancestral enolase shared by all mycoplasmas. However, separate functions of H7 have not been confirmed, and H7 was suggested to be related to interactions with host molecules in our previous study (Chen et al., 2019). We also found a partial motif of H7 in the primary sequences of enolases from species of other genera of Mollicutes as mentioned above. However, there is not enough structural information to support this view.

The oligomeric states of enolases, especially the enolases of prokaryotes, have always been inconclusive. With the development of structural biology, the oligomeric states of enolases from various species have been further verified by the determined structures (Lu et al., 2012; Chen et al., 2019). Generally, dimeric enolases cannot be transformed to octamers by any symmetry operation (Lu et al., 2012). However, octameric enolases can easily be returned to their original state by a simple symmetry operation. According to the analysis of Mb Eno and Mp Eno interfaces, it is clear that octameric interface was formed by other amino acids rather than those of dimeric interface (Table 1). Here, we collected enolases from different species and found that all eukaryotic enolases are dimers and that most prokaryotic enolases are octamers (Supplementary Table S2). *S. aureus* enolase is an exception, as it adopts both dimeric and octameric oligomer states (Wu et al., 2015). However, only octameric *S. aureus* enolase has full enolase enzyme activity, which indicates that the octameric isoform is the functional form. A study shows that coexisting of monomers, dimers, and octamers has been observed in enolase from *Trichomonas vaginalis*. The ratio of three types of oligomers is variable depended on protein concentration and cosolute type (Mirasol-Meléndez et al., 2018). However, both of Mb Eno and Mp Eno seem to have predominant oligomer states in limited conditions studied here. Some clues are also found from the interface areas (Table 1). All of the oligomer interface areas of Mb Eno or Mp Eno are bigger than the corresponding ones of enolase from *Streptococcus suis*, which populate only the octameric state (Lu et al., 2012). This indicates the oligomer states of Mb Eno and Mp Eno may be stable. Three genes encode mammalian enolase: α-, β- and ϒ-enolase (Rider and Taylor, 1974; Rider and Taylor, 1975; Fletcher et al., 1976). However, Eubacteria and Archaebacteria have only a single enolase gene (Macías-Sánchez et al., 2015). Mammal enolase genes are thought to have evolved by gene duplication and mutation from an ancestral gene (Innan and Kondrashov, 2010). Archaeal enolases and most eubacterial enolases are octamers, indicating that ancestral enolase may adopt an octameric state. Ancestral eukaryotic enolases likely evolved into dimers in the early stage of the phylogenetic tree and then remained relatively stable. Dimeric enolases from a few bacteria, such as *E. coli*, *Coxiella burnetii* and *M. pneumoniae*, are thought to have experienced independent evolution after separation from their ancestors.

In conclusion, our study will also help improve the understanding of the independent evolution of *Mycoplasma* species, even those of Mollicutes.

## Data Availability

The atomic coordinates for Mb Eno and Mp Eno have been deposited in the PDB under IDs 7E2P and 7E2Q, respectively. The datasets presented in this study can be found in online repositories. The names of the repository/repositories and accession number(s) can be found in the article/Supplementary Material.
