# Supplementary material for: Evidence for the Rapid and Divergent Evolution of Mycoplasmas: Structural and Phylogenetic Analysis of Enolases
Source: Front Mol Biosci. 2022 Jan 25;8:811106. doi: 10.3389/fmolb.2021.811106 (PMC8822174; doi:10.3389/fmolb.2021.811106)
Supplement: Supplementary file 2 [file DataSheet6.PDF]

Supplementary information

Supplementary Figure 1

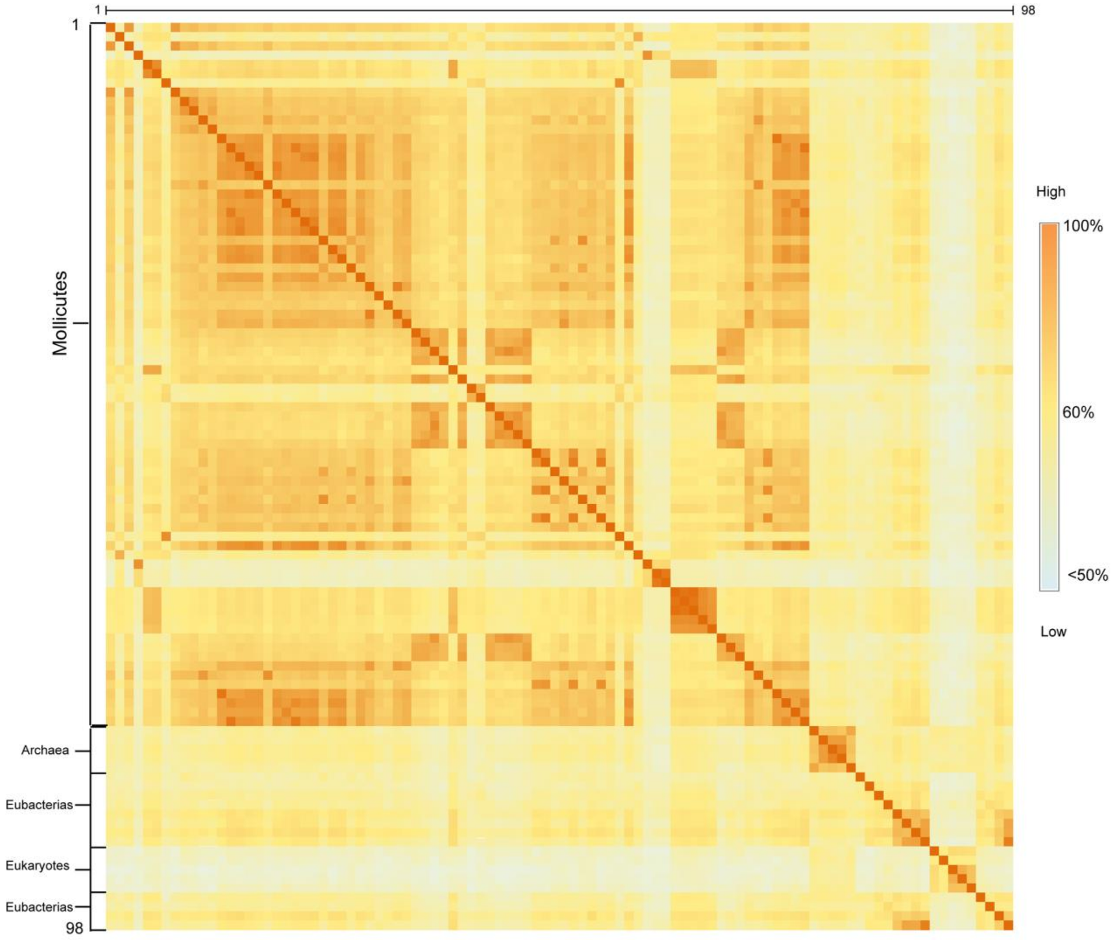

Fig. S1 Sequence similarity matrix for enolases from 98 species.

It is the heat map of the sequence identity matrix for enolases from 98 species. The types of the organisms, in which enolases are from, are noted at the left of the image. There are total of 98 species. 1-76 are enolases from Mollicutes. Others are labeled accordingly. The block shows the sequence identity between the enolases of the two intersecting species. The degree of sequence similarity is colored from dark orange to light blue. The numerical results can be found in Dataset S1.

Supplementary Figure 2

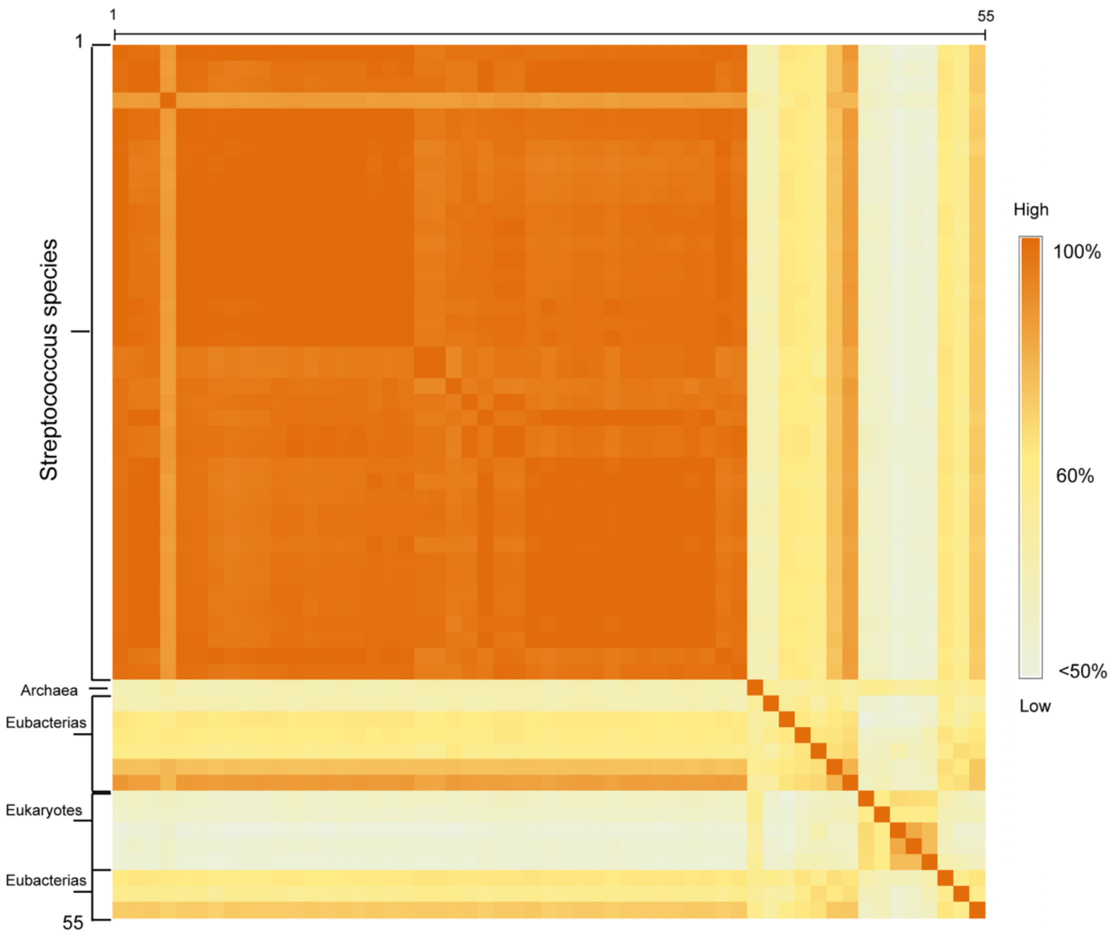

Fig. S2 Sequence similarity matrix between two enolases from different Streptococccus species.

It is the heat map of the sequence identity matrix for enolases from 40 streptococccus species. The types of the organisms, in which enolases are from, are noted at the left of the picture. There are total of 55 species. 1-40 are enolases from streptococccus species. Others are labeled accordingly. The block shows the sequence identity between the enolases of the two intersecting species. The degree of sequence similarity is colored from dark orange to light blue. The numerical results can be found in Dataset S2.

Supplementary Figure 3

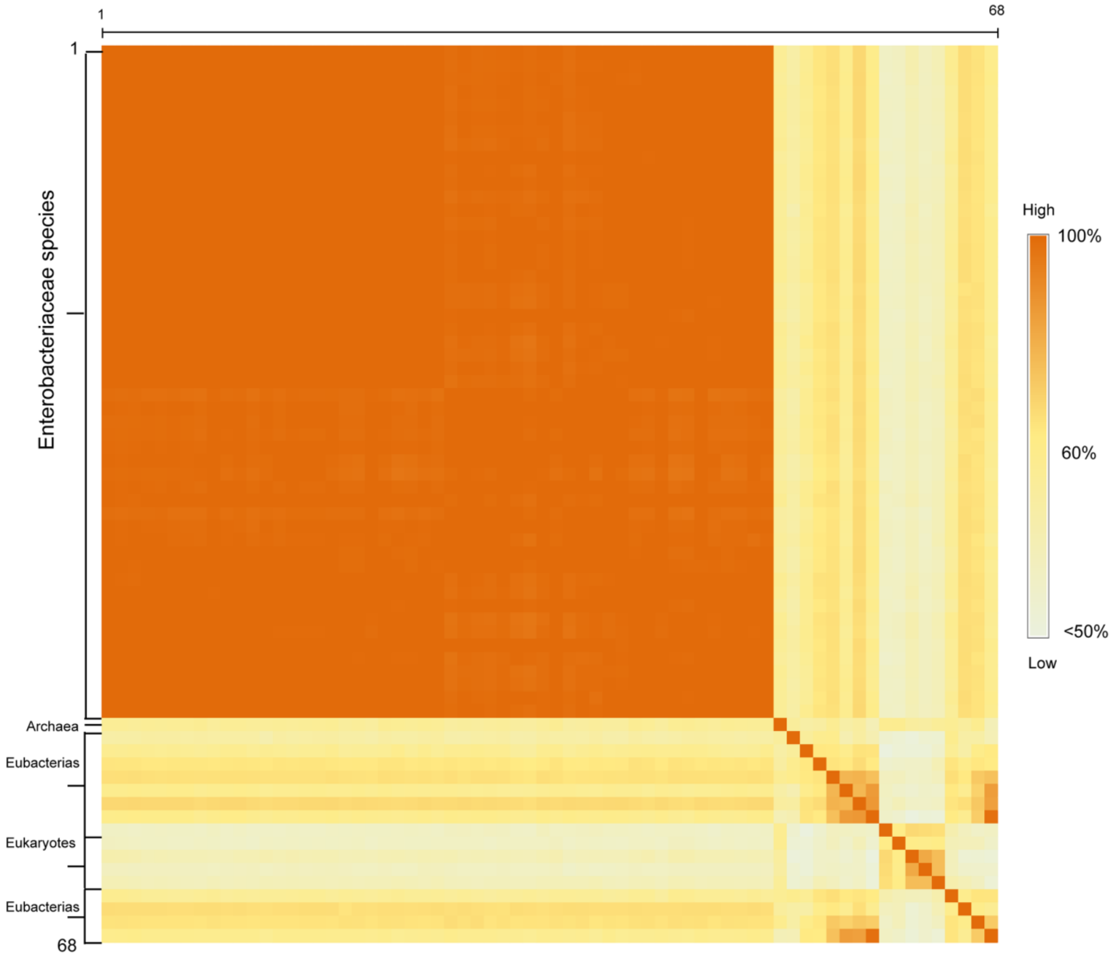

Fig. S3 Sequence similarity matrix between two enolases from different Enterobacteriaceae species.

It is the heat map of the sequence identity matrix for enolases from 51 enterobacteriaceae species. The types of the organisms, in which enolases are from, are noted at the left of the picture. There are total of 68 species.1-51 are enolases from enterobacteriaceae species. Others are labeled accordingly. The block shows the sequence identity between the enolases of the two intersecting species. The degree of sequence similarity is colored from dark orange to light blue. The numerical results can be found in Dataset S3.

Supplementary Figure 4

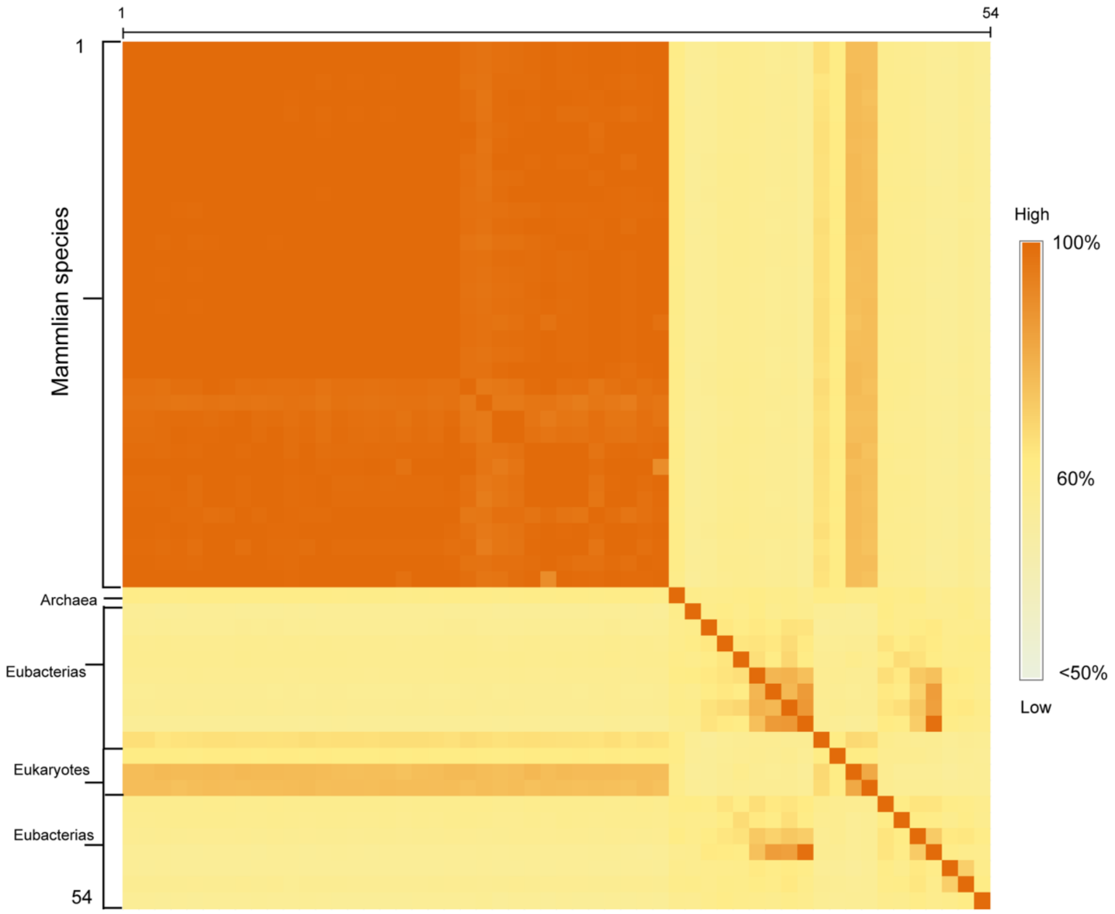

Fig. S4 Sequence similarity matrix between two enolases from different Mammlian species.

It is the heat map of the sequence identity matrix for enolases from 34 mammlian species. The types of the organisms, in which enolases are from, are noted at the left of the picture. There are total of 54 species. 1-34 are enolases from mammlian species. Others are labeled accordingly. The block shows the sequence identity between the enolases of the two intersecting species. The degree of sequence similarity is colored from dark orange to light blue. The numerical results can be found in Dataset S4.

Supplementary Figure 5

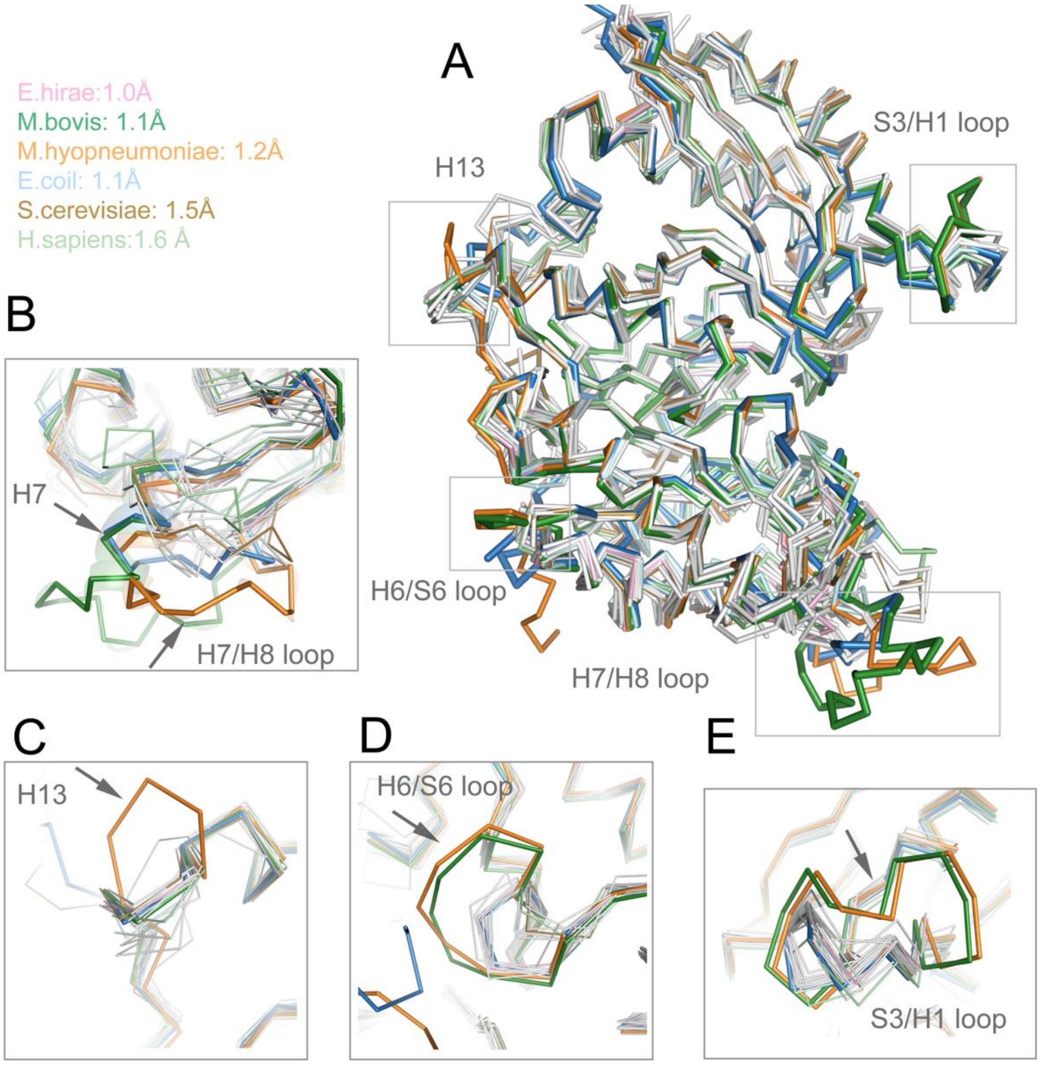

Fig. S5 Structural comparisons of mycoplasma enolases and other enolases.

Mp Eno is shown in blue. Mhp Eno is shown in bright orange. Mb Eno is shown in green. Enolases from humans, yeast, *E. coli* and *Enterococcus hirae* are shown in light green, light blue, brown and pink, respectively, and other enolases are shown in white. Structural deviations between Mp Eno and other enolases are indicated. An overall structural superimposition is shown in (A). (B–F) are enlarged pictures of the H7/H8 loop, H13, H6/S6 loop, and S3/H1 loop regions. Helix 7 is shown in both cartoon and ribbon forms in (B).

Supplementary Figure 6

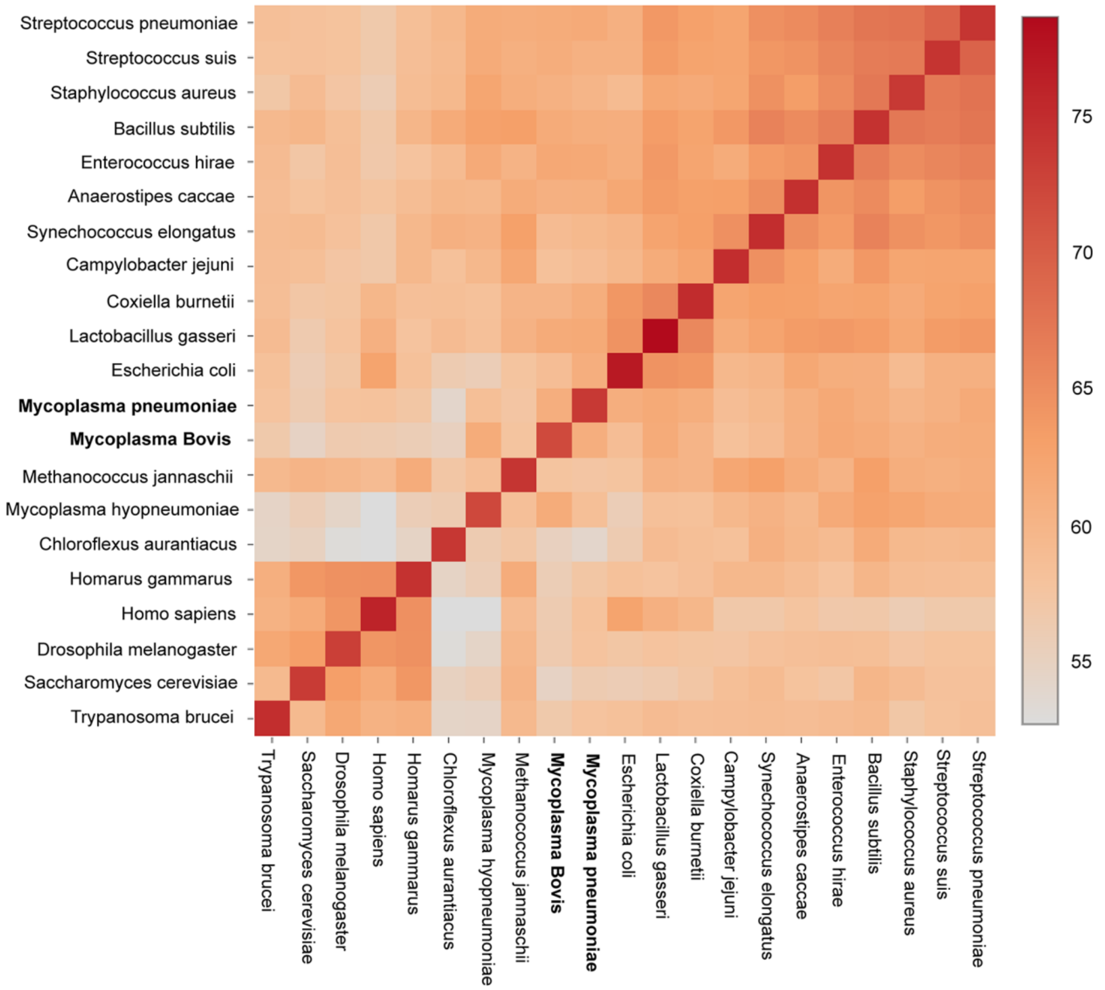

Fig. S6 Heat map of Z-scores for two different resolved enolase strctures.

Heat map of the structure identity matrix between two enolases from different species. The species names for every row and column are noted. The block shows the sequence identity between the enolases of the two intersecting species. The degree of sequence similarity is colored from red to white. PDB accession numbers are the same as used in Table S2.

Supplementary Figure 7

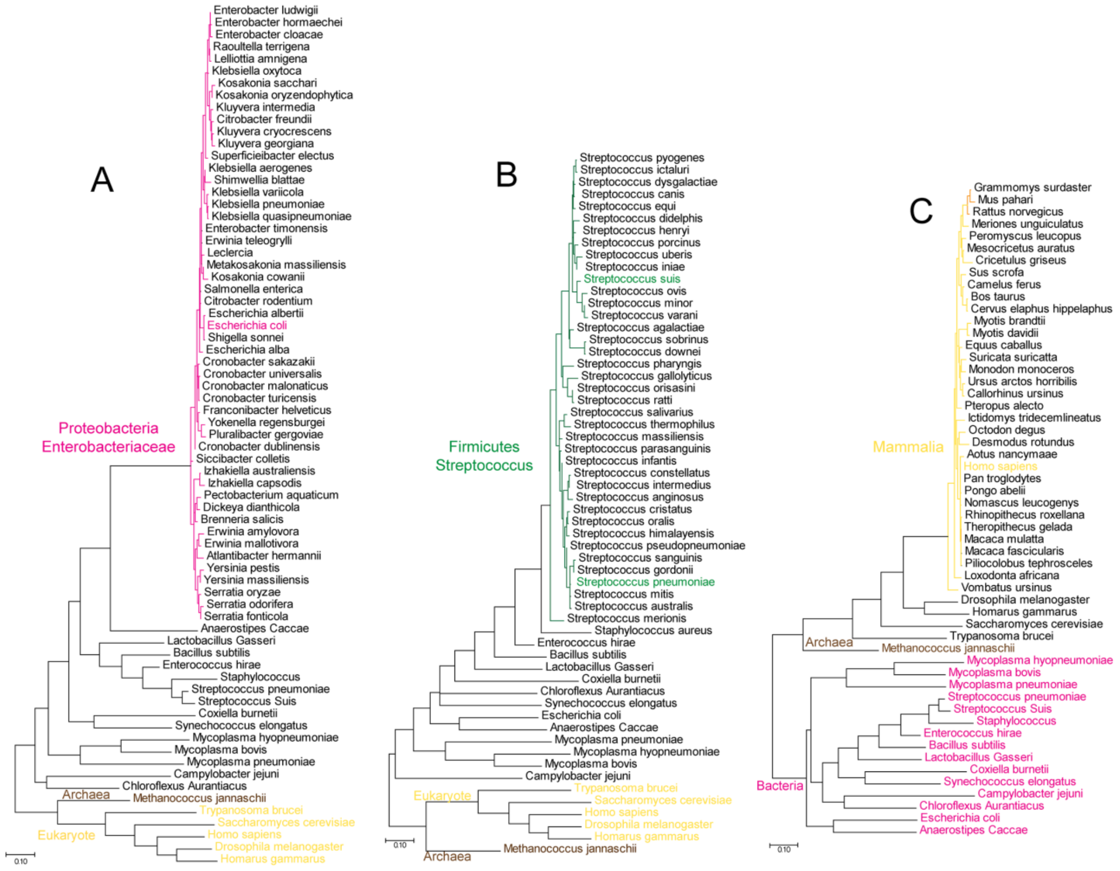

Fig. S7 Phylogenetic trees generated by using enolases from Enterobacteriaceae, Streptococcus and Mammalia.

(A, B and C) Evolutionary trees for Enterobacteriaceae, Streptococcus and mammals, respectively. *E. coli*, *S. suis*, *S. pneumoniae* and *H. sapiens*, whose enolase structures have been solved, are highlighted.

Supplementary Figure 8

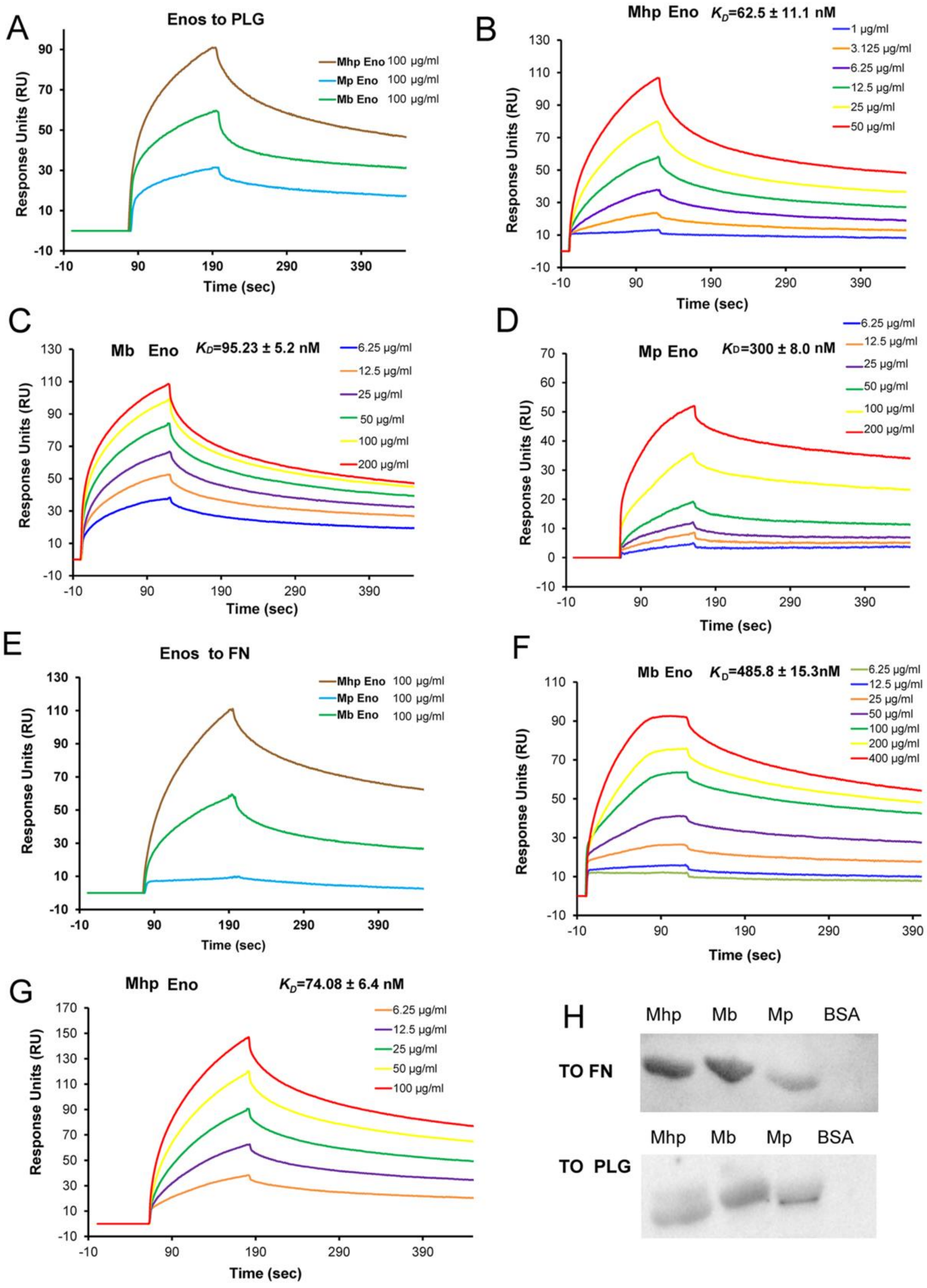

Fig. S8 Three different mycoplasma enolases have different affinities for PLG and FN.

(A-G) Enolases from *Mycoplasma bovis*, *M. pneumoniae* and *M. hyopneumoniae* at a

concentration gradient from 1 μg/ml to 200 μg/ml were flowed through immobilized PLG or FN in the SPR assay. The binding profiles are shown. (B) Mhp Eno binding to PLG. (C) Mb Eno binding to PLG. (D) Mp Eno binding to PLG. (F) Mb Eno binding to FN. (G) Mhp Eno binding to FN. (A and E) An integrated view comparing the binding response units of the indicated enolase proteins to PLG and FN (100 μg/ml). (H) Analysis of the interactions of mycoplasma enolase with FN and PLG by far WB. The protein names are indicated above the lanes. The interacting protein names are to the left of the pictures. BSA was used as the control protein.

Dataset S1. Sequence similarity matrix between two enolases from different species.

Dataset S2. Sequence similarity matrix between two enolases from different Streptococcccus species.

Dataset S3. Sequence similarity matrix between two enolases from different Enterobacteriaceae species.

Dataset S4. Sequence similarity matrix between two enolases from different Mammlian species.

Dataset S5. Mycoplasma genome information

Accession numbers for different enolases:

Mycoplasma hyopneumoniae, PDB ID: 6j36; *Mycoplasma flocculare,* WP_002557541.1; *Mycoplasma ovipneumoniae,* WP_069097229.1; *Mycoplasma*

bovoculi, WP_022935145.1; *Mycoplasma conjunctivae*, WP_012751597.1; *Mycoplasma molare*, WP_027123150.1; *Mycoplasma hyorhinis*, WP_013302289.1; *Mycoplasma anatis*, WP_006886432.1; *Mycoplasma glycophilum*, WP_027333875.1; *Mycoplasma bovirhinis*, BBA22262.1; *Mycoplasma sturni*, WP_036463757.1; *Mycoplasma pullorum*, WP_073372313.1; *Mycoplasma collis*, WP_033161261.1; *Mycoplasma columborale*, WP_036433871.1; *Mycoplasma alligatoris*, WP_005683930.1; *Mycoplasma buteonis*, WP_036452465.1; *Mycoplasma felis*, WP_036430823.1; *Mycoplasma canis*, WP_004794172.1; *Mycoplasma iners*, WP_029512513.1; *Mycoplasma leonicaptivi*, WP_027121045.1; *Mycoplasma cynos*, WP_015286902.1; *Mycoplasma gallinarum*, WP_027332606.1; *Mycoplasma synoviae*, WP_011283175.1; *Mycoplasma bovis*, WP_013456550.1; *Mycoplasma pulmonis*, WP_010925319.1; *Mycoplasma mobile*, WP_011264700.1; *Mycoplasma agalactiae*, WP_013021986.1; *Mycoplasma fermentans*, WP_013354454.1; *Mycoplasma hyosynoviae*, WP_036444825.1; *Mycoplasma salivarium*, WP_024544112.1; *Mycoplasma arginine*, WP_004415334.1; *Mycoplasma orale*, WP_022936242.1; *Spiroplasma eriocheiris*, WP_047791726.1; *Mycoplasma arthritidis*, WP_012498535.1; *Mycoplasma genitalium*, WP_009885616.1; *Mycoplasma pneumoniae*, PFH44449.1; *Mycoplasma cloacale*, WP_029330105.1; *Mycoplasma alkalescens*, WP_002880986.1; *Mycoplasma arginine*, WP_004415334.1; *Mycoplasma canadense*, WP_045433746.1; *Mycoplasma auris*, WP_004425037.1; *Mycoplasma salivarium*, WP_024544112.1; *Mycoplasma spumans*, WP_027122521.1; *Mycoplasma elephantis*, WP_027333991.1; *Mycoplasma simbae*,

WP_029608949.1; *Mycoplasma columbinum,* WP_006608730.1; *Mycoplasma lipofaciens,* WP_027120880.1; *Mycoplasma bovigenitalium,* WP_004421476.1; *Mycoplasma meleagridis*, WP_046096770.1; *Mycoplasma testudineum,* WP_094254493.1; *Mycoplasma californicum,* WP_038561377.1; *Mycoplasma opalescens*, WP_029905365.1; other mycoplasma enolase were from mycoplasma genome (Dataset S1.) *Pyrococcus horikoshii,* WP_010886009.1; *Palaeococcus pacificus,* WP_048164867.1; *Thermococcus barophilus,* WP_013467817.1; *Methanotorris formicicus,* EHP87376.1; *Methanococcus jannaschii,* PDB ID: 2PA6; *Campylobacter jejuni,* PDB ID: 3QN3; *Coxiella burnetii,* PDB ID: 3TQP; *Synechococcus elongatus,* PDB ID: 4ROP; *Escherichia coli* PDB ID: 1E9L; *Bacillus subtilis*, PDB ID: 4A3R; *Staphylococcus aureus,* PDB ID: 5BOF; *Enterococcus hirae*, PDB ID: 1LYX; *Streptococcus pneumonia*, PDB ID: 1W6T; *Saccharomyces cerevisiae* PDB ID: 3ENL; *Trypanosoma brucei*, PDB ID: 1OEP; *Drosophila melanogaster* PDB ID: 5WRO; *Homarus gammarus* PDB ID: 1PDZ; and *Homo sapiens,* PDB ID: 3B97.

Accession numbers for different EF-TU:

Mycoplasma hyopneumoniae, WP_011284269.1; *Mycoplasma pulmonis*, WP_129690203.1; *Mycoplasma collis*, WP_033161486.1; *Mycoplasma molare*, WP_027123418.1; *Mycoplasma bovoculi*, WP_022934762.1; *Mycoplasma conjunctivae,* WP_041594541.1; *Mycoplasma hyorhinis,* WP_020104673.1; *Mycoplasma ovipneumoniae*, WP_010321121.1; *Mycoplasma californicum*,

WP_129722737.1; *Mycoplasma lipofaciens*, WP_027120927.1; *Mycoplasma elephantis*, WP_027333968.1; *Mycoplasma leonicaptivi*, WP_027121215.1; *Mycoplasma anatis*, WP_006886429.1; *Mycoplasma alligatoris*, WP_005683895.1; *Mycoplasma alligatoris*, WP_005683895.1; *Mycoplasma columborale*, WP_036434301.1; *Mycoplasma felis*, WP_036430197.1; *Mycoplasma bovirhinis*, WP_099309096.1; *Mycoplasma sturni*, WP_036464832.1; *Mycoplasma canis*, WP_004795211.1; *Mycoplasma cynos*, WP_015287681.1; *Mycoplasma synoviae*, WP_011283803.1; *Mycoplasma buteonis*, WP_036452077.1; *Mycoplasma genitalium*, WP_009885583.1; *Mycoplasma pneumoniae*, WP_054175792.1; *Mycoplasma mobile*, WP_011264744.1; *Mycoplasma salivarium*, WP_024544157.1; *Mycoplasma hyosynoviae*, WP_036441217.1; *Mycoplasma testudineum*, WP_094254298.1; *Mycoplasma orale*, WP_022935742.1; *Mycoplasma canadense*, WP_045434054.1; *Mycoplasma iners*, WP_029512648.1; *Mycoplasma auris*, WP_111518246.1; *Mycoplasma agalactiae*, WP_101456848.1; *Mycoplasma* spumans, WP_027122526.1; *Mycoplasma columbinum*, WP_006608829.1; *Mycoplasma arginine*, WP_004415386.1; *Mycoplasma bovigenitalium*, WP_004418907.1; *Mycoplasma gallinarum*, WP_027332905.1; *Mycoplasma fermentans*,VEU60285.1; *Mycoplasma meleagridis*, WP_046097128.1; *Mycoplasma pullorum*, WP_073372484.1; *Mycoplasma glycophilum*, WP_027333868.1; *Mycoplasma cloacale*, WP_029330109.1; *Mycoplasma alkalescens*, WP_002880918.1; *Mycoplasma bovis*, AMW25710.1; *Mycoplasma simbae*, WP_029608780.1, *Mycoplasma arthritidis*, WP_012498300.1; *Spiroplasma eriocheiris*, WP_047790972.1; *Spiroplasma*

helicoides, WP_069117434.1; *Spiroplasma culicicola*, WP_025363567.1; *Mycoplasma gallisepticum*, WP_011113468.1 ; *Mycoplasma ovis*, WP_024070985.1; *Homo sapiens*, AAB00499.1; *Bacillus subtilis*, AUS12345.1; *Saccharomyces cerevisiae* YJM1083, AJT87299.1; *Escherichia coli*, WP_000031795.1; *Trypanosoma brucei brucei* TREU927, XP_827821.1; *Coxiella burnetii*, WP_080708104.1; *Synechococcus*, WP_011242968.1; *Enterococcus hirae*, WP_135981547.1; *Staphylococcus aureus*, MVG43597.1; *Streptococcus pneumoniae*, WP_088788320.1; *Campylobacter jejuni* subsp. jejuni 414, EFC33002.1; *Methanocaldococcus fervens*, WP_015791815.1; *Methanocaldococcus jannaschii*, WP_010869821.1. *Archaeoglobi archaeon*, TDA30282.1; *Euryarchaeota archaeon*, NOZ58619.1; *Geoglobus ahangari*, WP_048095085.1

Accession numbers for different 16S rRNAs

Mycoplasma collis, NR_041930.1; *Mycoplasma molare*, NR_114637.1; *Mycoplasma iners*, NR_025064.1; *Mycoplasma conjunctivae*, NR_044781.1; *Mycoplasma pullorum*, NR_026017.1; *Mycoplasma simbae*, NR_025964.1; *Mycoplasma leonicaptivi*, NR_025965.1; *Mycoplasma alligatoris*, NR_041844.1; *Mycoplasma ovipneumoniae*, NR_025989.1; *Mycoplasma gallinarum*, NR_113687.1; *Mycoplasma testudineum*, NR_044767.1; *Mycoplasma buteonis*, NR_025177.1; *Mycoplasma lipofaciens*, NR_025065.1; *Mycoplasma glycophilum*, NR_025184.1; *Mycoplasma elephantis*, NR_025071.1; *Mycoplasma spumans*, NR_113678.1; *Mycoplasma opalescens*, NR_025067.1; *Mycoplasma hyosynoviae*, NR_029183.1; *Mycoplasma*

elephantis, NR_025071.1; *Mycoplasma bovirhinis*, NR_025986.1; *Mycoplasma canadense*, NR_025988.1; *Ureaplasma parvum*, NR_074762.2; *Spiroplasma eriocheiris*, NR_125505.1; *Ureaplasma gallorale*, NR_026027.1; *Ureaplasma urealyticum*, NR_041710.1; *Spiroplasma syrphidicola*, NR_025711.1; *Spiroplasma helicoides*, NR_025704.1; *Bacillus subtilis*, NC_000964.3; *Coxiella burnetii*, NZ_CP013667.1; *Escherichia coli*, NZ_CP027599.1; *Synechococcus elongates*, NZ_CP033061.1; *Enterococcus hirae*, NC_018081.1; *Staphylococcus aureus*, NC_007795.1; *Streptococcus pneumoniae*, NC_003098.1; *Campylobacter jejuni*, NC_002163.1; *Methanocaldococcus jannaschii*, NC_000909.1; *Thermococcus barophilus*, CP002372.1; *Palaeococcus pacificus*, CP006019.1; *Ferroplasma acidiphilum*, CP015363.1

Accession numbers for different Enterobacteriaceae enolases

Shigella sonnei, WP_119170830.1; *Escherichia albertii*, WP_150003799.1; *Salmonella enterica*, EAA7621511.1; *Citrobacter rodentium*, WP_012907154.1; *Enterobacter timonensis*, WP_061707359.1; *Erwinia] teleogrylli*, WP_058913517.1; *Escherichia alba*, WP_155107017.1; *Franconibacter helveticus*, WP_024551277.1; *Klebsiella aerogenes*, WP_101705368.1; *Metakosakonia massiliensis*, WP_044180284.1; *Cronobacter sakazakii*, WP_004386867.1; *Shimwellia blattae*, WP_002442418.1; *Cronobacter sakazakii*, WP_054625615.1; *Cronobacter universalis*, WP_007701325.1; *Klebsiella aerogenes*, WP_063444489.1; *Cronobacter malonaticus*, WP_032979903.1; *Cronobacter*, WP_007763684.1; *Klebsiella*

pneumoniae, WP_117262187.1; *Enterobacter ludwigii*, AEW75015.1; E*nterobacter hormaechei*, WP_161503912.1; *Cronobacter dublinensis*, WP_105738703.1; *Klebsiella oxytoca*, WP_154681753.1; *Citrobacter freundii*, WP_130715417.1; *Enterobacter cloacae*, WP_063160921.1; *Klebsiella quasipneumoniae*, WP_101994709.1; *Superficieibacter electus*, WP_103675226.1; *Yersinia pestis*, WP_016678559.1; *Pectobacterium aquaticum*, WP_116155118.1; *Yersinia massiliensis*, WP_049604766.1; *Serratia odorifera*, WP_004955312.1; *Serratia oryzae*, WP_156292658.1; *Izhakiella australiensis*, WP_078003179.1; *Izhakiella capsodis*, WP_092875840.1; *Serratia fonticola*, WP_142010008.1; *Kosakonia sacchari*, SEL15901.1; *Erwinia amylovora*, WP_004168701.1; *Erwinia mallotivora*, WP_034938499.1; *Atlantibacter hermannii*, WP_002434624.1; *Brenneria salicis*, WP_113868108.1; *Dickeya dianthicola*, WP_161142984.1; *Kosakonia oryzendophytica*, WP_061497448.1; *Kluyvera intermedia*, WP_062775478.1; *Siccibacter colletis*, WP_031522861.1; *Kluyvera cryocrescens*, WP_052282129.1; *Kluyvera Georgiana*, WP_064543089.1; *Yokenella regensburgei*, WP_006818494.1; *Kosakonia cowanii*, WP_139568348.1; *Raoultella terrigena*, WP_045857537.1; *Lelliottia amnigena*, WP_064327201.1; *Pluralibacter gergoviae*, WP_045289323.1; *Klebsiella variicola*, WP_110242777.1.

Accession numbers for different mammalian enolases

Pan troglodytes, NP_001207708.1; *Suricata suricatta*, XP_029803093.1; *Bos Taurus*, NP_776474.2; *Monodon monoceros*, XP_029085258.1; *Mesocricetus auratus*,

XP_021088233.1; *Camelus ferus*, XP_032351011.1; *Equus caballus*, NP_001254532.1; *Sus scrofa*, XP_020950937.1; *Ursus arctos horribilis*, XP_026375696.1; *Octodon degus*, XP_004644277.1; *Pteropus alecto*, XP_006914449.1; *Aotus nancymaae*, XP_012329038.1; *Rhinopithecus roxellana*, XP_010362492.1; *Macaca fascicularis*, NP_001270890.1; *Pongo abelii*, NP_001126461.1; *Piliocolobus tephrosceles*, XP_023071038.1; *Theropithecus gelada*, XP_025215442.1; *Macaca mulatta*, NP_001182540.1; *Nomascus leucogenys*, XP_003274363.1; *Callorhinus ursinus*, XP_025711778.1; *Cricetulus griseus*, EGW01873.1; *Vombatus ursinus*, XP_027699638.1; *Myotis brandtii*, EPQ12763.1; *Myotis davidii*, ELK28033.1; *Meriones unguiculatus*, XP_021504228.1; *Peromyscus leucopus*, XP_028714285.1; *Rattus norvegicus*, NP_001103378.1; *Grammomys surdaster*, XP_028613777.1; *Desmodus rotundus*, XP_024409965.1; *Ictidomys tridecemlineatus*, XP_005334801.1; *Mus pahari*, XP_021056757.1; *Loxodonta Africana*, XP_003413233.1; *Cervus elaphus hippelaphus*, OWK08279.1.

Accession numbers for different Steptococcus enolases

Streptococcus pneumoniae, PDB: 1W6T_A; *Streptococcus pyogenes*, PDB: 3ZLH_A; *Staphylococcus aureus*, PDB: 5BOE_A; *Streptococcus mitis*, WP_153196746.1; *Streptococcus australis*, WP_006595950.1; *Streptococcus salivarius*, WP_155211692.1; *Streptococcus anginosus*, WP_106384375.1; *Streptococcus constellatus*, WP_006269397.1; *Streptococcus intermedius*, WP_125364084.1; *Streptococcus himalayensis*, WP_068991889.1; *Streptococcus massiliensis*,

WP_018372298.1; *Streptococcus cristatus*, WP_005589720.1; *Streptococcus parasanguinis,* WP_118227983.1; *Streptococcus oralis,* WP_125847471.1; *Streptococcus infantis* , WP_006151882.1; *Streptococcus sanguinis,* WP_149566368.1; *Streptococcus pseudopneumoniae,* WP_138707612.1; *Streptococcus gordonii*, WP_046165366.1; *Streptococcus sobrinus*, WP_019785646.1; *Streptococcus downei*, WP_115325026.1; *Streptococcus merionis*, WP_018372816.1; *Streptococcus gallolyticus*, WP_012962183.1; *Streptococcus ovis*, WP_018378290.1; *Streptococcus orisasini*, WP_057491612.1; *Streptococcus ratti*, WP_003087713.1; *Streptococcus uberis*, WP_037627481.1; *Streptococcus minor*, WP_018167568.1; *Streptococcus didelphis*, WP_018365763.1; *Streptococcus porcinus*, WP_138068317.1; *Streptococcus iniae*, WP_003100050.1; *Streptococcus varani*, WP_093651113.1; *Streptococcus dysgalactiae*, WP_012766766.1; *Streptococcus henryi*, WP_018164428.1; *Streptococcus canis*, WP_164228831.1; *Streptococcus ictaluri*, WP_008089274.1; *Streptococcus agalactiae*, WP_161515274.1; *Streptococcus equi*, WP_012515409.1; *Streptococcus thermophiles*, WP_011225708.1; *Streptococcus pharynges*, WP_146566376.1.
