# Supplementary material for: Evidence for the Rapid and Divergent Evolution of Mycoplasmas: Structural and Phylogenetic Analysis of Enolases
Source: Front Mol Biosci. 2022 Jan 25;8:811106. doi: 10.3389/fmolb.2021.811106 (PMC8822174; doi:10.3389/fmolb.2021.811106)
Supplement: Supplementary file 4 [file Table5.DOCX]

| Species | Dimer or Octamer | PDB Code |
| --- | --- | --- |
| *Trypanosoma brucei* | Dimer | 1OEP |
| *Homarus gammarus* | Dimer | 1PDZ |
| *Homo sapiens* | Dimer | 3B97 |
| *Saccharomyces cerevisiae* | Dimer | 3ENL |
| *Drosophila melanogaster* | Dimer | 5WRO |
| *Escherichia coli* | Dimer | 1E9I |
| *Coxiella burnetii* | Dimer | 3TQP |
| *Mycoplasma pneumoniae* | Dimer | 7E2Q |
| *Bacillus subtilis* | Octamer | 4A3R |
| *Mycoplasma bovis* | Octamer | 7E2P |
| *Mycoplasma hyopneumoniae* | Octamer | 6J36 |
| *Enterococcus hirae* | Octamer | 1IYX |
| *Streptococcus pneumoniae* | Octamer | 1W6T |
| *Methanococcus jannaschii* | Octamer | 2PA6 |
| *Campylobacter jejuni* | Octamer | 3QN3 |
| *Synechococcus elongatus* | Octamer | 4ROP |
| *Chloroflexus aurantiacus* | Octamer | 4YWS |
| *Staphylococcus aureus* | Octamer | 5BOF |
| *Streptococcus suis* | Octamer | 4EWJ |
| *Lactobacillus gasseri* | Octamer | 4MKS |
| *Anaerostipes caccae* | Octamer | 3UJ2 |

**Table S2. Oligomeric states of different enolases.**
