# Supplementary material for: Evidence for the Rapid and Divergent Evolution of Mycoplasmas: Structural and Phylogenetic Analysis of Enolases
Source: Front Mol Biosci. 2022 Jan 25;8:811106. doi: 10.3389/fmolb.2021.811106 (PMC8822174; doi:10.3389/fmolb.2021.811106)
Supplement: Supplementary file 5 [file Table4.DOCX]

|  | **1** | **2** | **3** | **4** | **5** | **6** | **7** | **8** | **9** | **10** | **11** | **12** | **13** | **14** | **15** | **16** | **17** | **18** | **19** | **20** | **21** |
| --- | --- | --- | --- | --- | --- | --- | --- | --- | --- | --- | --- | --- | --- | --- | --- | --- | --- | --- | --- | --- | --- |
| **1** | **74.8** | 59.2 | 61.9 | 60.4 | 60.8 | 54.4 | 54.5 | 59.3 | 56.7 | 57.8 | 58.2 | 59 | 58.6 | 58.7 | 58.9 | 58.7 | 59 | 59.3 | 57.1 | 58 | 58.4 |
| **2** | 59.2 | **73.4** | 63.1 | 61.4 | 64 | 55 | 55.7 | 60 | 54.7 | 56.2 | 55.9 | 56.4 | 57.2 | 58.5 | 59.1 | 57.9 | 57.2 | 59.8 | 59 | 58.1 | 58.1 |
| **3** | 61.9 | 63.1 | **73** | 64.1 | 64.6 | 53 | 54.4 | 59.6 | 56.4 | 57.8 | 57.3 | 57.8 | 57.5 | 57.4 | 58.1 | 58.4 | 58.6 | 58.5 | 57.4 | 57.9 | 57.9 |
| **4** | 60.4 | 61.4 | 64.1 | **75.9** | 64.7 | 52.7 | 52.7 | 59 | 56.2 | 58 | 62.5 | 60.6 | 59.7 | 56.9 | 56.9 | 57.7 | 56.8 | 56.9 | 56 | 56.7 | 56.7 |
| **5** | 60.8 | 64 | 64.6 | 64.7 | **74.4** | 54.6 | 55.7 | 61.2 | 55.8 | 57.3 | 58.2 | 57.8 | 58.4 | 59.5 | 59.5 | 58.8 | 57.8 | 59.8 | 58.8 | 58.6 | 58.5 |
| **6** | 54.4 | 55 | 53 | 52.7 | 54.6 | **73.9** | 56.2 | 57.2 | 55.1 | 54.1 | 56.2 | 59 | 58.5 | 58.2 | 60.6 | 59.7 | 59.1 | 61.4 | 59.4 | 59.2 | 59.5 |
| **7** | 54.5 | 55.7 | 54.4 | 52.7 | 55.7 | 56.2 | **72.1** | 58.4 | 61.3 | 58.5 | 55.7 | 58.3 | 58.2 | 59.5 | 60.3 | 59.5 | 61.5 | 62.7 | 62.2 | 61.4 | 61.3 |
| **8** | 59.3 | 60 | 59.6 | 59 | 61.2 | 57.2 | 58.4 | **74.1** | 57.7 | 57.4 | 57.7 | 60.3 | 60 | 62 | 62.9 | 61.3 | 60.2 | 63 | 61 | 60.7 | 61.1 |
| **9** | 56.7 | 54.7 | 56.4 | 56.2 | 55.8 | 55.1 | 61.3 | 57.7 | **71.9** | 60.9 | 58.7 | 61.4 | 60 | 58.2 | 59 | 60.6 | 61.8 | 61.4 | 60.5 | 61.1 | 61.3 |
| **10** | 57.8 | 56.2 | 57.8 | 58 | 57.3 | 54.1 | 58.5 | 57.4 | 60.9 | **73.7** | 60.9 | 61.6 | 61 | 58.7 | 59.4 | 60.7 | 61.7 | 61 | 59.9 | 60.5 | 61.5 |
| **11** | 58.2 | 55.9 | 57.3 | 62.5 | 58.2 | 56.2 | 55.7 | 57.7 | 58.7 | 60.9 | **77** | 64.4 | 64 | 59.5 | 59.9 | 61.9 | 61 | 60.9 | 59.1 | 60.5 | 60.6 |
| **12** | 59 | 56.4 | 57.8 | 60.6 | 57.8 | 59 | 58.3 | 60.3 | 61.4 | 61.6 | 64.4 | **78.5** | 65.5 | 61.3 | 62.4 | 63.4 | 63.7 | 63.3 | 61.9 | 63.4 | 63.8 |
| **13** | 58.6 | 57.2 | 57.5 | 59.7 | 58.4 | 58.5 | 58.2 | 60 | 60 | 61 | 64 | 65.5 | **75** | 62.3 | 63 | 62.9 | 62.3 | 62.5 | 61.5 | 62.5 | 62.8 |
| **14** | 58.7 | 58.5 | 57.4 | 56.9 | 59.5 | 58.2 | 59.5 | 62 | 58.2 | 58.7 | 59.5 | 61.3 | 62.3 | **74.9** | 64.7 | 63 | 61.3 | 63.8 | 62.2 | 62.3 | 62.3 |
| **15** | 58.9 | 59.1 | 58.1 | 56.9 | 59.5 | 60.6 | 60.3 | 62.9 | 59 | 59.4 | 59.9 | 62.4 | 63 | 64.7 | **74.9** | 64.8 | 63.5 | 66.1 | 64.6 | 64 | 64.7 |
| **16** | 58.7 | 57.9 | 58.4 | 57.7 | 58.8 | 59.7 | 59.5 | 61.3 | 60.6 | 60.7 | 61.9 | 63.4 | 62.9 | 63 | 64.8 | **74.6** | 64.2 | 65.2 | 63.2 | 64.4 | 65.2 |
| **17** | 59 | 57.2 | 58.6 | 56.8 | 57.8 | 59.1 | 61.5 | 60.2 | 61.8 | 61.7 | 61 | 63.7 | 62.3 | 61.3 | 63.5 | 64.2 | **74.4** | 66.5 | 65.1 | 65.7 | 66.3 |
| **18** | 59.3 | 59.8 | 58.5 | 56.9 | 59.8 | 61.4 | 62.7 | 63 | 61.4 | 61 | 60.9 | 63.3 | 62.5 | 63.8 | 66.1 | 65.2 | 66.5 | **74.4** | 67.1 | 66.7 | 67.4 |
| **19** | 57.1 | 59 | 57.4 | 56 | 58.8 | 59.4 | 62.2 | 61 | 60.5 | 59.9 | 59.1 | 61.9 | 61.5 | 62.2 | 64.6 | 63.2 | 65.1 | 67.1 | **73.6** | 66.9 | 67.7 |
| **20** | 58 | 58.1 | 57.9 | 56.7 | 58.6 | 59.2 | 61.4 | 60.7 | 61.1 | 60.5 | 60.5 | 63.4 | 62.5 | 62.3 | 64 | 64.4 | 65.7 | 66.7 | 66.9 | **74.2** | 69.3 |
| **21** | 58.4 | 58.1 | 57.9 | 56.7 | 58.5 | 59.5 | 61.3 | 61.1 | 61.3 | 61.5 | 60.6 | 63.8 | 62.8 | 62.3 | 64.7 | 65.2 | 66.3 | 67.4 | 67.7 | 69.3 | **74.1** |

Table S1. Z-score matrix for two different resolved enolase structures.

| Enolase structures from 1: *Trypanosoma brucei*; 2: *Saccharomyces cerevisiae*; 3: *Drosophila melanogaster*; 4: *Homo sapiens*; 5: *Homarus gammarus*; 6: *Chloroflexus aurantiacus*; 7: *Mycoplasma hyopneumoniae*;8: *Methanococcus jannaschii*; 9: *Mycoplasma Bovis*; 10: *Mycoplasma pneumoniae*; 11: *Escherichia coli*; 12: *Lactobacillus gasseri* 13: *Coxiella burnetii*; 14: *Campylobacter jejuni*: 15: *Synechococcus elongatus*; 16: *Anaerostipes caccae* ;17: *Enterococcus hirae*;18: *Bacillus subtilis*; 19: *Staphylococcus aureus*; 20: *Streptococcus suis*; 21: *Streptococcus Pneumoniae*. |
| --- |
